# *Ocimum sanctum* Alters the Lipid Landscape of the Brain Cortex and Plasma to Ameliorate the Effect of Photothrombotic Stroke in a Mouse Model

**DOI:** 10.3390/life13091877

**Published:** 2023-09-07

**Authors:** Inderjeet Yadav, Nupur Sharma, Rema Velayudhan, Zeeshan Fatima, Jaswinder Singh Maras

**Affiliations:** 1 National Brain Research Centre, Gurugram 122052, India; inderjeet@nbrc.ac.in (I.Y.); rema.velayudhan@gmail.com (R.V.); 2 Department of Medical Laboratory Sciences, College of Applied Medical Sciences, University of Bisha, Bisha 61922, Saudi Arabia; 3 Department of Molecular and Cellular Medicine, Institute of Liver and Biliary Sciences, New Delhi 110070, India; nupush1995@gmail.com; 4 Amity Institute of Biotechnology, Amity University Haryana, Gurugram 122413, India

**Keywords:** lipidomic profile, Tulsi, *Ocimum sanctum*, ischemic stroke, photothrombotic ischemia, medicinal plant

## Abstract

Stroke-like injuries in the brain result in not only cell death at the site of the injury but also other detrimental structural and molecular changes in regions around the stroke. A stroke-induced alteration in the lipid profile interferes with neuronal functions such as neurotransmission. Preventing these unfavorable changes is important for recovery. *Ocimum sanctum* (Tulsi extract) is known to have anti-inflammatory and neuroprotective properties. It is possible that Tulsi imparts a neuroprotective effect through the lipophilic transfer of active ingredients into the brain. Hence, we examined alterations in the lipid profile in the cerebral cortex as well as the plasma of mice with a photothrombotic-ischemic-stroke-like injury following the administration of a Tulsi extract. It is also possible that the lipids present in the Tulsi extract could contribute to the lipophilic transfer of active ingredients into the brain. Therefore, to identify the major lipid species in the Tulsi extract, we performed metabolomic and untargeted lipidomic analyses on the Tulsi extract. The presence of 39 molecular lipid species was detected in the Tulsi extract. We then examined the effect of a treatment using the Tulsi extract on the untargeted lipidomic profile of the brain and plasma following photothrombotic ischemic stroke in a mouse model. Mice of the C57Bl/6j strain, aged 2–3 months, were randomly divided into four groups: (i) Sham, (ii) Lesion, (iii) Lesion plus Tulsi, and (iv) Lesion plus Ibuprofen. The cerebral cortex of the lesioned hemisphere of the brain and plasma samples were collected for untargeted lipidomic profiling using a Q-Exactive Mass Spectrometer. Our results documented significant alterations in major lipid groups, including PE, PC, neutral glycerolipids, PS, and P-glycerol, in the brain and plasma samples from the photothrombotic stroke mice following their treatment with Tulsi. Upon further comparison between the different study groups of mice, levels of MGDG (36:4), which may assist in recovery, were found to be increased in the brain cortexes of the mice treated with Tulsi when compared to the other groups (*p* < 0.05). Lipid species such as PS, PE, LPG, and PI were commonly altered in the Sham and Lesion plus Tulsi groups. The brain samples from the Sham group were specifically enriched in many species of glycerol lipids and had reduced PE species, while their plasma samples showed altered PE and PS species when compared to the Lesion group. LPC (16:1) was found in the Tulsi extract and was significantly increased in the brains of the PTL-plus-Tulsi-treated group. Our results suggest that the neuroprotective effect of Tulsi on cerebral ischemia may be partially associated with its ability to regulate brain and plasma lipids, and these results may help provide critical insights into therapeutic options for cerebral ischemia or brain lesions.

## 1. Introduction

Cerebral ischemia is one of the leading causes of all human strokes [1]. It results in neuronal death, primarily in the ischemic region, and neuronal degeneration in the penumbral region [2]. It is the first leading cause of disability and the second leading cause of mortality worldwide, accounting for about 6,000,000 deaths annually [3]. The World Health Organization (WHO) speculates that there will be over 7.8 million stroke-linked deaths per year by 2030 [4]. A stroke occurs due to an inadequate supply of oxygen to parts of the brain, either due to a disrupted supply of blood (ischemic stroke) to some parts of the brain or sometimes due to a sudden breach in a blood vessel in the brain (hemorrhagic stroke). So, if the blood supply in part of the brain is interrupted, it results in neuronal death and neuronal degeneration, due to which symptoms of cerebral ischemia can be seen in the body. 

Many published studies have reported a link between dyslipidemia and stroke [5,6,7]. Dyslipidemia, mostly hyperlipidemia, is an abnormal amount of blood lipids. An increase in blood lipid levels could be a predisposing factor for the pathogenesis of stroke, mainly ischemic stroke. Previous reports in the literature have suggested a strong association between stroke and lipid metabolism [8,9,10,11]. Lipid profile assessments in brain anomalies could be helpful as lipids are considered the main constituents of the brain. Lipids comprise 60% of its dry weight and represent the second-most abundant tissue in the brain after adipose tissue [12]. They are essentially required for maintaining the physiological functions of the neurons and also aid in their structural development. Thus, understanding the dynamics of lipid molecules through lipidomics could help interpret altered brain functions in stroke. Although significant advances have been made in understanding stroke pathogenesis in recent years, studies focusing on brain and plasma lipidomics have been limited [13,14,15]. 

Ayurvedic medicine is one of the ancient systems of medicine [16] in which many natural and herbal compounds are used to cure human diseases because of their medicinal properties. One such plant, *Ocimum sanctum* (Tulsi), has been very well known as a medicinal herb since ancient times. It has many medicinal properties and has been reported to possess minimal to zero adverse side effects [17,18]. *Ocimum sanctum*, commonly known as holy basil or “Tulsi”, is called the “Elixir of Life” for its healing powers and is frequently used as a medicinal agent in the Ayurvedic and Siddha medical systems to ameliorate numerous body ailments [19,20,21]. Tulsi has been used for decades for its potential to treat a number of diseases, including anxiety, cough, asthma, diarrhea, fever, dysentery, arthritis, eye diseases, otalgia, indigestion, hiccups, vomiting, gastric, cardiac, and genitourinary disorders, back pain, skin diseases, ringworm, insect, snake, and scorpion bites, and malaria [22,23,24,25], due to its broad spectrum of pharmacological activities. It has been found to have diverse protective effects, including hepato-protective, immuno-modulatory, anti-ulcer, anti-diabetic, anti-hypercholesterolemic, nerve tonic, chemo-protective, nootropic, antitussive, anti-inflammatory, wound healing, anti-tumorigenesis, anti-convulsant, anthelmintic, anti-bacterial, anti-anxiety, and anti-stress activities [26,27,28,29,30].

However, thus far, the effects of Tulsi treatments on cerebral ischemia are limited [31]. The oral administration of an aqueous extract of Tulsi for 15 days before MCAO demonstrated a marked reduction in infarct size, reduced neurological deficits, and suppressed neuronal loss in MCAO rats [31]. Pretreatment with a methanolic extract of Tulsi for 7 days significantly prevented cerebral-hypoperfusion-induced functional and structural disturbances and was useful in the treatment of cerebral reperfusion injury and cerebrovascular insufficiency states [27]. Tulsi has demonstrated anti-inflammatory effects in animal models of acute and chronic inflammation [32]. Additionally, nanostructured lipid carriers of a Tulsi leaf extract were shown to inhibit both the Cox-1 and Cox-2 enzyme pathways, highlighting the potent anti-inflammatory potential of Tulsi and its compounds [33]. Tulsi was shown to be useful for the management of experimentally-induced cognitive dysfunctions in rats [32]. The effect of Tulsi treatment following ischemia on alterations of the lipid profile of the brain and plasma is not known. Also, there is a lack of information on whether there are changes in the lipid species in the brain with the progression of stroke.

In the current study, lipidomic profiles of the brain and plasma samples of animals that had undergone photothrombotic stroke were evaluated following treatment with Tulsi. Lipid changes in the brain cortex and plasma of mice were analyzed using liquid chromatography coupled with mass spectrometry (LC-MS). Lipid changes associated with a change in stroke pathophysiology due to the healing effect of Tulsi were identified and reported. In addition, the change in brain lipid composition was correlated to that seen in the plasma samples of lesioned animals that were treated with Tulsi. The identified lipids could be used as a clinical indicator of brain recovery and could also be exploited as therapeutic targets.

## 2. Material and Methods

### 2.1. Animals

For this study, 24 male mice of the C57Bl/6j strain aged 2–3 months were procured from the animal house of the National Brain Research Centre, Gurgaon, India. All the experimental procedures were duly approved by the Institutional Animal Ethics Committee (IAEC) of the National Brain Research Center. Animals were housed in standard cages with dimensions of 44 × 29 × 16 cm (LXWXH). They were maintained under controlled environmental conditions with a temperature maintained at 22 ± 1 °C, relative humidity between 45 and 55%, a 12:12 h light-dark cycle, and 12–15 air changes per hour as specified in CPCSEA (Committee for the Purpose of Control and Supervision of Experiments on Animals, GOI) guidelines. Pelleted feed procured from Altromin, Germany, and autoclaved water were provided *ad libitum* to all the animals. 

### 2.2. Experimental Groups

The animals were randomly divided into four groups: (i) Sham; (ii) PTL only; (iii) PTL plus Tulsi; (iv) PTL plus Ibu.

Sham animals (*n* = 8) were the controls for the experiment. They underwent all surgical procedures similar to all other animals but were not subjected to Photothrombotic Lesion (PTL). They were anesthetized, and the skin above the skull was incised. The skull was exposed, and subsequently, the skin was sutured. An antibiotic cream, Neosporin, was applied to the wound. Brains and plasma from four animals in this group were used for lipidomic analyses. The brain sections of four animals were used as controls for determining the extent of PTL lesions using cytochrome oxidase staining.

PTL-only animals (*n* = 8) were subjected to PTL. Animals in this group had focal unilateral photothrombotic lesions made by exposing the cortex to laser for 10 min after injecting Rose Bengal red dye (I.V). Four animals from this group were used for the brain and plasma lipidome analyses. Brain sections from four animals were analyzed for cytochrome oxidase to determine the extent of the lesion.

PTL plus Tulsi (*n* = 4) animals were subjected to PTL, and for seven days post-PTL, Tulsi leaf extract was orally administered to them. Tulsi ethanolic extract (Batch Number U/1443/17-18) was purchased from M/S Saiba Industries PVT. Ltd., Mumbai, India. The extract was dissolved in sterile water at a concentration of 60 mg/mL. Each animal was given oral gavage of 500 mg/kg body weight daily. We selected the dosage based on studies showing beneficial effects at this concentration [34,35,36]. Studies examining the toxicity of *Ocimum sanctum* [37,38] have shown no adverse effects at this concentration. 

PTL plus Ibuprofen animals (*n* = 4) were subjected to PTL, and subsequently, for seven days, they were given a 100 mg/kg oral dose of Ibuprofen (Cipla) oral syrup [39,40].

### 2.3. Photothrombotic Lesion (PTL)

The surgical area was sterilized using 70% alcohol and betadine, and all surgical instruments were sterilized by autoclaving before the surgery. Animals were anesthetized with a mixture of ketamine (100 mg/kg body weight) and xylazine (10 mg/kg body weight) given through the I/P route. The photothrombotic cortical lesion was made in mice’s brains as described by Watson et al. (1985) [41] with few modifications. The head of the animal was firmly secured on the stereotaxic apparatus by inserting the ear bars carefully into the external meatus to avoid any damage to the eardrum, and a midline incision was made from eye level down to the neck using a scalpel. Skin retractors were applied to keep the skull exposed. The Bregma and lambda were the landmarks for stereotaxic coordinates. The somatosensory cortex, 0.7 mm posterior from the bregma and 2.8 mm lateral to the midline (2.5 mm diameter), was marked with a marker pen. An area of about 2.5 mm in diameter, which includes a large part of the somatosensory cortex according to the mouse brain atlas by Franklin and Paxinos [42] on the left hemisphere, was marked. A sterilized black plastic paper with a hole of 2.5 mm diameter was placed on the skull such that the hole was above the marking of the somatosensory cortical region while the other regions were covered. The body temperature of the animals was maintained at 37 ± 0.5 °C during the surgery with the help of a heating pad.

Rose Bengal solution 20 mg/kg was given as a slow intravenous injection through the tail vein. All sources of light in the room were turned off. A green laser (532 nm laser irradiation, 50 mW/cm^2^) was switched on for 10 min. Following laser exposure, the skin of the head region was sutured. The mouse was removed from the stereotaxic apparatus and placed on a heating pad (pre-warmed) until it became fully awake. It was then returned to its home cage.

### 2.4. Cytochrome Oxidase Reaction

**Figure 1:**
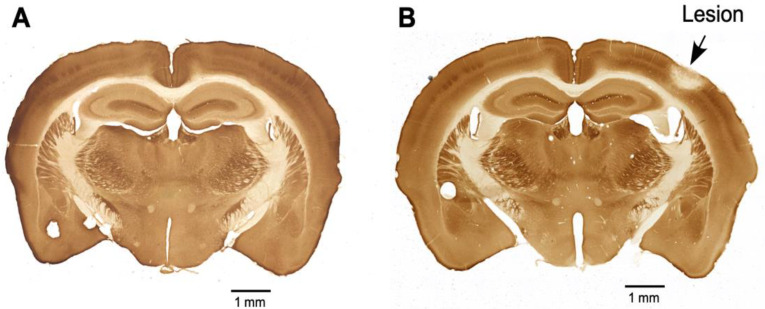
Photomicrograph showing brain sections that were reacted for cytochrome oxidase from (**A**) a sham control mouse and (**B**) a PTL mouse. The lesioned area, indicated by the arrow, shows a region with inactive cells.

The four animals with sham control and the four animals with PTL were given saline for seven days post-lesion. On the eighth day, the animals were perfused transcardially with PBS (NaCl (80 gm), KCl (2 gm), Na_2_HPO_4_ (11.4 gm), and KH_2_PO_4_ (23.2 g) in 900 mL of water and then forming a volume of 1 L, pH-7.4) to clear the blood and then with 4% paraformaldehyde in PBS (PFA) to fix the tissues. The brains were removed and postfixed for 24 h in 10% sucrose in a PFA solution at 4 °C. The brains were cryoprotected by sequentially allowing them to sink in 20% sucrose in PBS, followed by 30% sucrose in PBS at 4 °C. The forebrain was cut into 30 µm thick sections on a sliding microtome. Every sixth section was reactive for cytochrome oxidase. The sections were washed three times with PBS at room temperature and then immersed in freshly prepared cytochrome oxidase staining solution (in 30 mL of 0.1 M phosphate buffer, sucrose (5 g), cytochrome C (Sigma, St. Louis, MO, USA; 25 mg), and DAB (Sigma; 20 mg) were dissolved, and the volume of solution was made up to 50 mL with phosphate buffer). The sections were incubated for 5 h at 37 °C. The sections were then washed twice with PBS, mounted on gelatin-coated glass slides, air dried, and coverslipped with DPX mounting medium. The sections were observed under a light microscope and imaged. Figure 1 shows sections from the brains of sham control animals (Figure 1A) and PTL animals (Figure 1B). In Figure 1B, the cortical region where the photothrombotic lesion was done appears pale, suggesting that cells in the area are dead, as indicated by the absence of cytochrome oxidase activity. 

### 2.5. Sample Collection and Preparation

**Figure 2:**
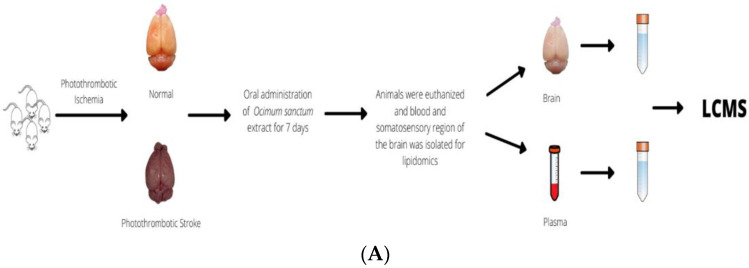

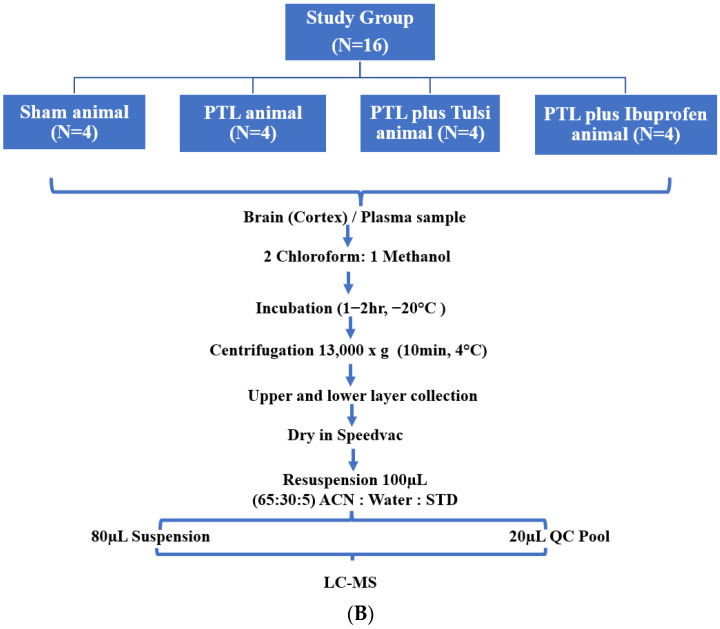
(**A**) The overall scheme for the untargeted lipidomic analysis of brain and plasma. (**B**) Workflow to perform lipidomic analysis.

Twenty-four hours following the last treatment dose, samples were collected for lipidome analyses. Blood (600 μL) from the heart of each animal was collected after cervical dislocation and kept on ice. The animals were then decapitated, and the lesioned hemisphere was rapidly removed and flash-frozen in liquid nitrogen. Blood samples were centrifuged at 3000 rpm for 15 min at 4 °C, and plasma (the supernatant) was collected. Both brain and plasma samples were stored at −80 °C until lipid extraction. Figure 2A shows the schematic diagram of the procedure for untargeted lipidomic analyses of the brain and plasma.

Brain (cortex) and plasma samples were prepared for lipidomic analysis as described in Sharma N et al., 2022 [43]. Briefly, 100 µL brain/plasma samples were added to a chloroform: methanol (2:1) mixture and homogenized. After that, the samples were centrifuged at 13,000 rpm for 10 min to extract the dissolved lipids (supernatant). Each sample was then vacuum-dried. These dried samples were reconstituted in 100 μL of a 65:30:5:5 standard solution consisting of acetonitrile (65%): isopropanol (30%): water (5%): internal and external standards (5%). Using an ultra-high-performance liquid chromatographic system, they were subjected to reverse-phase chromatography in the C18 column (Thermo Scientific™25003102130: 3 µm, 2.1 mm, 100 mm). Mobile phase A was 0.1% formic acid, and mobile phase B was 100% acetonitrile. The sample processing flow chart is shown in Figure 2B.

### 2.6. Mass Spectrometry

For performing mass spectroscopy, a 10 µL sample was passed through the column, which was directed into the heated electrospray ionization (HESI) source of a Q-Exactive mass spectrometer (Thermo Scientific, San Jose, CA, USA), and analysis was carried out in both positive and negative ionization modes in two independent runs. The HESI source parameters were as follows: The spray voltage was set to 3.7 kV in positive ionization mode and −3.1 kV in negative ionization mode. The heated capillary temperature was maintained at 360 °C, and the sheath and auxiliary gas flow were set to 15 and 10 (arbitrary units), respectively. All the samples were pooled together and spiked with internal standards (Dinose b: 1 mg/mL; MCPA: 1 mg/mL; Dimetrazole: 1 mg/mL) and external standards (Cholesterol: 0.1 mg/mL; Colchicine: 4 mg/mL; Impramine: 2 mg/mL; Roxithromycin: 2 mg/mL; Amiloride: 1 mg/mL; Atropine: 2 mg/mL; 2-aminoanthroceae: 370.5 mg/mL; Prednisolone: 2 mg/mL) to make dilutions of 1:1, 1:2, 1:4 and 1:8 which were operated as QC for the higher-energy collisional dissociation (HCD) MS/MS experiment. This study used metabolite internal and external standards to attain analytical sensitivity for the MS/MS experiments. In MS/MS mode, the isolation width was set to *m*/*z* 1.5, the normalized collision energy was 32%, and the mass resolution was set at 17,500 FWHM at *m*/*z* 200. 

#### Mass Spectrometry for Metabolomics of Tulsi Extract

Mass spectrometry for metabolomics of Tulsi extract was performed using a 100 µL sample of plant extract and chilled methanol in a 1:4 ratio (400 µL methanol). The sample was incubated at −20 °C for 10 h or overnight. After incubation, the sample was centrifuged at 13,000 rpm for 10 min, and the supernatant was taken and discarded in the pellet. The supernantant was freeze dried completely, and the dried sample was reconstituted in 100 μL of 90:5:5 reconstitution buffer (90% water, 5% acetonitrile, 5% external standard and internal standard). The sample was run for mass spectrometry as described by Sharma et al. [43].

Composition of internal standards (Dinose b: 5 μg/mL; MCPA (2 methyl-4-chlorophenoxy acetic acid): 5 μg/mL; Dimetrazole: 5 μg/mL, AMPA: 5 μg/mL) and external standards (Dihydrostreptomycin: 20 μg/mL; Colchicine: 0.5 μg/mL; Impramine: 0.5 μg/mL; Roxithromycin: 10 μg/mL; Amiloride: 10 μg/mL; Atropine: 1 μg/mL; 2-aminoanthroceae: 1 μg/mL; Prednisolone: 1 μg/mL; Metformin: 1 μg/mL; Ethylmalonic acid: 3 μg/mL). 

### 2.7. Software Analysis

Lipid features were identified using LipidSearch 4.0 software (Thermo Scientific, San Jose, CA, USA). The feature identification and quantitation parameters used are mentioned in Supplementary File S2.

### 2.8. Statistical Analysis

Annotated lipid features were subjected to different statistical software platforms. First, missing value imputation was applied to data in which half of the minimum positive value was estimated for lipids that were undetected in the samples. Subsequently, data were filtered based on non-parametric relative standard deviation (MAD/median) and subjected to log normalization and Pareto-scaled using the Metaboanalyst 5.0 server “http://metaboanalyst.ca” (17 October 2022) [44]. Unpaired (two-tail) Student’s *t*-test and the Mann–Whitney U test were performed for comparison of two groups. For more than two groups, one-way ANOVA (analysis of variance), and the Kruskal–Wallis test were performed. PCA, PLS-DA, heat map, random-forest analysis, and other statistical analyses were performed. Venn diagram analysis was utilized to understand the correlation between brain and plasma lipid profiles, and *p*-values of <0.05 using Benjamini-Hochberg correction were considered statistically significant.

## 3. Results

This study examined the effect of oral administration of Tulsi for seven days on the lipid profile of the cerebral cortex of the brain and the plasma of mice with ischemic lesions induced by photothrombosis. Untargeted lipidomic analysis was performed on the cerebral cortex of the brain and also on the blood plasma. We have analyzed changes associated with lipid molecules in the lesioned brain cortex of mice following oral administration of Tulsi for seven days. One of the study’s objectives was to determine whether the changes induced by Tulsi were similar to those induced by a known anti-inflammatory drug, Ibuprofen, which was also administered orally for seven days. A comparison of lipid profiles between animal groups with sham, PTL, PTL plus Tulsi, and PTL plus Ibuprofen-treated animals was performed to determine whether treatment with Tulsi could restore the normal lipid profile in lesioned animals.

### 3.1. Tulsi Modulates Lipidomic Signature in the Lesioned Cortical Hemisphere of Mice with Photothrombotic Ischemic Stroke-like Lesion

**Figure 3:**
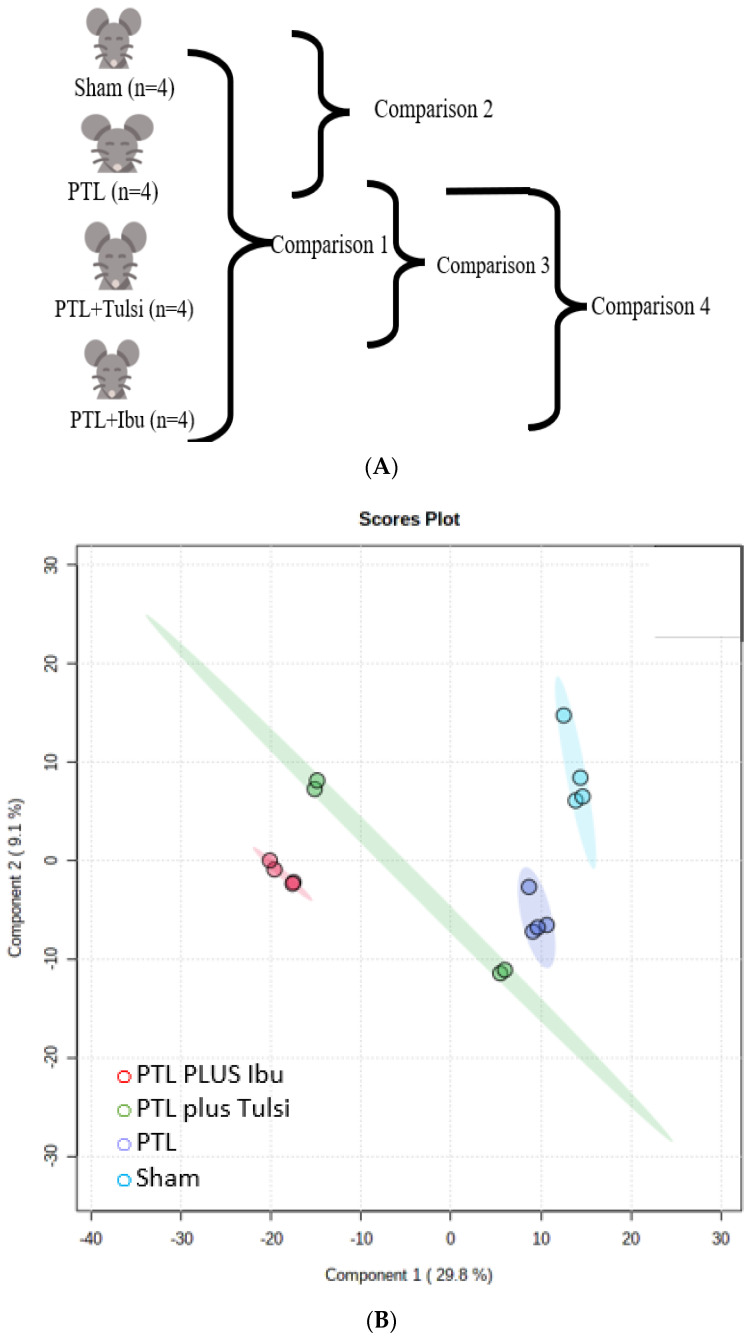

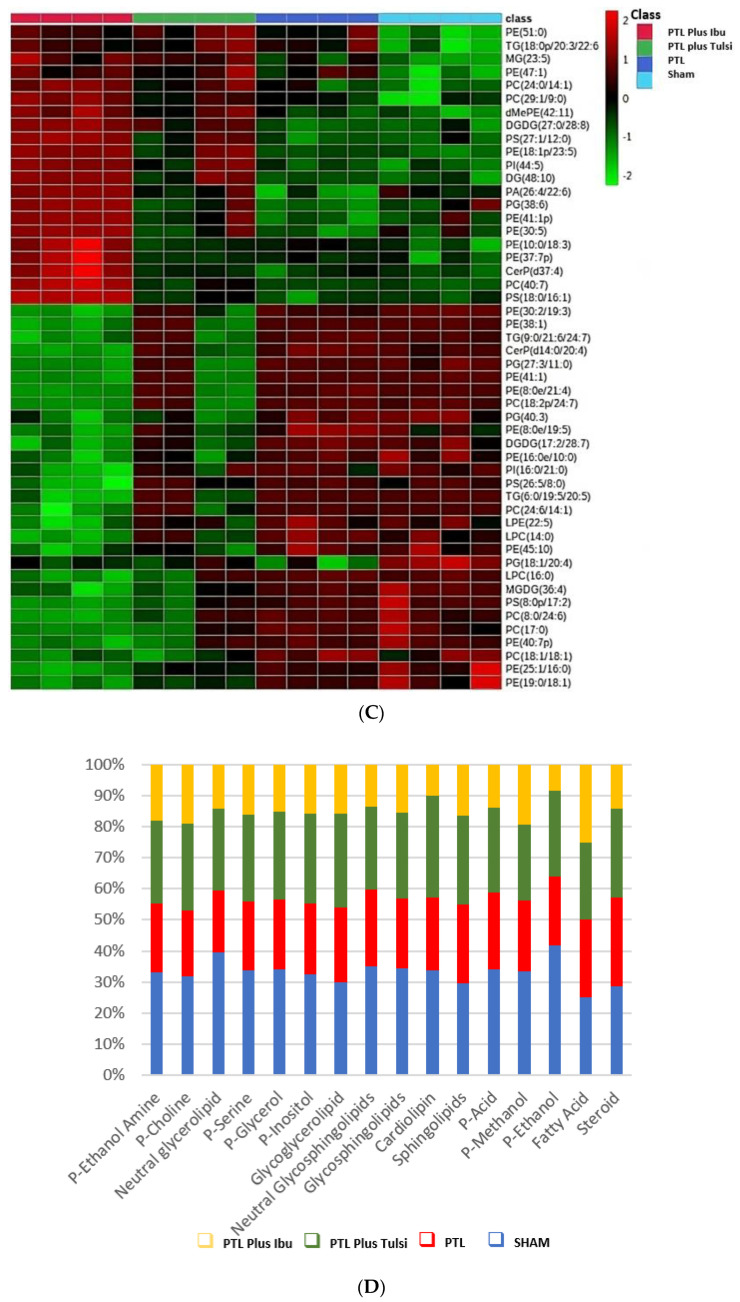

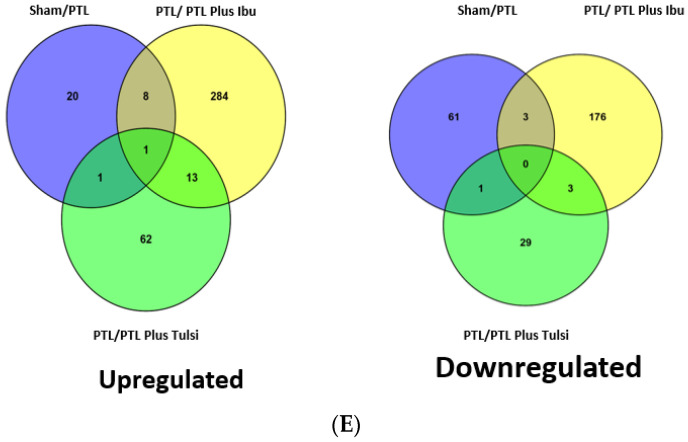
(**A**) The schematic representation of different comparisons (sham, PTL, PTL plus Tulsi, and PTL plus Ibu) for the brain and plasma is denoted by comparisons 1, 2, 3, and 4. (**B**) PLS−DA score plot of lipid species in the ipsilateral hemisphere of the brain cortex of the sham, PTL, PTL plus Tulsi, and PTL plus Ibu. (**C**) A heatmap showing the expression patterns of identified differential lipids in the brain from different comparison groups. Upregulated lipids are shown in red, while downregulated lipids are shown in green. (**D**) Relative abundance of different lipid groups in the ipsilateral hemisphere of the brain cortex of the Sham, PTL, PTL plus Tulsi, and PTL plus Ibu. (**E**) The common and unique upregulated and downregulated lipids are identified through the Venn diagram in the ipsilateral hemisphere of the brain cortex.

Untargeted lipidomic analysis was performed on cerebral cortex samples. The cerebral cortex of the left hemisphere of all the experimental animals was used for lipidomic profiling. Figure 3A illustrates the different types of comparisons done on the lipids of the cerebral cortex of experimental and sham-lesioned animals. Figure 3B demonstrates the results of PLS-DA analysis, highlighting the similarities and differences of the brain lipidome amongst the four study groups, i.e., sham, PTL, PTL plus Tulsi, and PTL plus Ibu.

The score plot with PC1 (29.8%) and PC2 (9.1%) clearly distinguished each group. Compact and distinct clustering was seen for Sham, PTL, PTL plus Ibu, and PTL plus Tulsi. The PTL plus Tulsi-treated mice group was distinctly positioned as compared to other groups; this observation suggested a critical role for Tulsi in the modulation of brain lipid profiles. Hierarchical clustering analysis was performed to better illustrate the differences in the lipid profile of the brain amongst the four groups (Figure 3C). A visual comparison of the lipids of the different groups in the heat map suggested that the PTL plus Tulsi animals have similarities in many of the upregulated as well as downregulated lipid species with PTL plus Ibu.

Hierarchical clustering analysis also showed that the lipidomic profile of the mice’s brains in the PTL plus Tulsi group was strikingly similar to that of the sham-operated group. These results again reconfirm that treatment with Tulsi normalizes the brain lipidomic profile. Multi-group random forest analysis of different lipid groups from the cortex of the four groups of animals identified 15 lipid species ranked by mean decrease in accuracy (Supplementary Figure S1).

Additional comparisons of alterations in the lipid profiles of the brain were performed between Sham vs. PTL, PTL vs. PTL plus Tulsi and PTL vs. PTL plus Ibu (Supplementary Figures S2–S10). A volcano map comparison (Supplementary Figure S2) of the lipids of brain samples from the sham vs. PTL group revealed 32 upregulated lipid molecules and 65 downregulated lipid molecules (log_2_ FC = 1.5 and *p* < 0.05). The PLS-DA analysis with a component variance of 17.8% (component 1) and 19.2% (component 2) separated the lipids from Sham and PTL into two distinct clusters (Supplementary Figure S3A). The VIP score from PLS-DA of lipid species in the brain of Sham vs. PTL has identified 35 lipid species (Supplementary Figure S3B). The heat map showed the differential upregulation and downregulation of lipids in the brains of Sham and PTL mice (Supplementary Figure S4A). Random forest modeling between lipid groups in the brain of Sham vs. PTL has identified 15 lipid species ranked by mean decrease in accuracy (Supplementary Figure S4B).

Treatment with Tulsi induced changes in the lipid composition in the brain of lesioned mice (PTL plus Tulsi) compared with PTL mice. Volcano map (Supplementary Figure S5) shows significant alteration of 110 lipid species (77 upregulated and 33 downregulated; log_2_ FC = 1.5 and *p* < 0.05). Segregation and distinct clustering of the PTL plus Tulsi group from the PTL groups were evident from PLS-DA analyses based on the component variance of 36.1% (PC1) and 14.3% (PC2), indicating significant differences in lipid composition between the two groups (Supplementary Figure S6A). The VIP score plot showed 35 lipid species that were highly modulated between PTL plus Tulsi and PTL groups (Supplementary Figure S6B). The upregulated and downregulated lipids in the brain of PTL plus Tulsi vs. PTL mice displayed in the heat map also showed that the lipids were differentially altered (Supplementary Figure S7A). Random forest modeling identified 15 species of lipids ranked by mean decrease in accuracy that differentially modulated brain lipid composition between PTL plus Tulsi and PTL (Supplementary Figure S7B).

We determined whether treatment with the non-steroidal anti-inflammatory drug Ibuprofen can affect the lipidome profile in the brains of mice with PTL by comparing PTL plus Ibu vs. PTL using the Volcano Map (Supplementary Figure S8). There were significant changes in 488 lipid species (306 upregulated and 182 downregulated; log_2_ FC = 1.5 and *p*-value < 0.05). PLS-DA revealed that the lipids from the brain of PTL plus Ibu mice form a distinct cluster well separated from the PTL cluster with a component variance of 47.1% (PC1) and 12.5% (PC2) (Supplementary Figure S9A). The plot presenting the VIP score showed the 35 most modified lipid species (Supplementary Figure S9B). A heat map of the lipids in the brains of PTL plus Ibu and PTL mice showed that there was differential regulation of lipid species such that the lipids that were downregulated in the PTL condition were upregulated in the PTL plus Ibu brains (Supplementary Figure S10A). Random forest modeling showed 15 species of lipids ranked by mean decrease in accuracy that differentially modulated brain lipid composition between PTL plus Ibu and PTL (Supplementary Figure S10B).

We also determined the relative abundance of lipid groups in the brains of the four groups of mice, i.e., Sham, PTL, PTL plus Tulsi, and PTL plus Ibu. (Figure 3D; Supplementary Table S1). When we examined the lipid profile of the cerebral cortex, we found that 16 groups were abundant in the brains of the four groups of mice. The relative abundance of these 16 lipid groups was compared among the brains of four different groups of mice to determine the effects of different treatments on lipid metabolism in the brain. 

The sham group exhibited the highest number of lipid species compared to the P-ethanolamine group. The relative abundance of phosphatidyl-choline, neutral glycerolipid, phosphatidyl-serine, phosphatidyl-glycerol, and phosphatidyl-inositol was also high in the brains of the four groups of animals. The results showed that the highest representation of 13 lipid groups was in the brains of sham group animals. This suggested that the normal brain lipid profile was different from that of lesioned animals. When comparing the brains of the PTL mice to the sham mice, it was found that the relative abundance of all 13 lipid groups was lower in the PTL group. This indicated that the PTL significantly affected lipid metabolism in the brain. 

When comparing the brains of the PTL plus Ibu mice to the PTL mice, it was found that the relative abundance of 13 lipid groups was increased in the PTL plus Ibu mice. However, the relative abundance of most lipid groups was still lower than that of the sham mice. This suggests that ibuprofen treatment was ineffective in fully restoring the normal lipid profile in the brains of lesioned animals. On the other hand, when comparing the brains of the PTL plus Tulsi mice to the PTL mice, it was found that the relative abundance of all 13 lipid groups was higher in the PTL plus Tulsi mice. In fact, all 13 lipid groups that were highest in the brains of sham mice were also higher in the brains of PTL plus Tulsi mice compared to PTL or PTL plus Ibu mice. This indicated that treatment with Tulsi effectively restored the normal lipid profile in the brains of lesioned animals. The significance of these observations was that Tulsi might possess the ability to reverse the deleterious effect of a lesion on the lipidome profile in the ischemic brain.

### 3.2. Comparison of Lipidome in Photothrombotic Ischemia-Induced Brain of Mice Treated with Tulsi vs. Ibuprofen

In order to find out the crucial lipids that were altered in photothrombotic lesions by Tulsi or Ibuprofen treatment, we used a Venn diagram (Figure 3E) to find out the lipid species that were commonly modulated in the brains of sham, PTL, PTL plus Tulsi, and PTL plus Ibu groups.

Venn analysis of upregulated lipids between sham/PTL, PTL/PTL plus Tulsi, and PTL/PTL plus Ibu showed only one lipid species, monogalactosyldiacylglycerol (36:4), to be commonly upregulated between these groups (Figure 3E: Supplementary Table S3). Its presence in response to the lesion and treatment with Tulsi or Ibuprofen indicated that a significant increase in MGDG (36:4) lipid might play a role in the transition from pathological to physiological conditions in brain samples. This lipid could be an important marker to determine the injury-induced reaction in the brain. 

When the 77 lipid species that were upregulated in brains of PTL /PTL plus Tulsi were compared with the 306 lipid species upregulated in PTL/PTL plus Ibu there were 13 common upregulated lipid species, dimethyl-phosphatidylethanolamine (18:1/14:0), phosphatidyl-ethanolamine (16:0/22:4), sphingomyelin (d44:7), phosphatidylcholine (17:0), phosphatidyl-ethanolamine (43:10), monogalactosyl-monoacylglycerol (14:2), di-galactosyl-diacylglycerol (42:9), phosphatidylinositol (28:5), phosphatidylserine (8:0e/21:6), lyso-phosphatidylcholine (16:1), phosphatidyl-ethanolamine (39:6), di-galactosyl-diacylglycerol (45:9) and diglyceride (42:5e) (Figure 3E: Supplementary Table S3).

When downregulated lipid species were compared between PTL/PTL plus Tulsi (33 lipid species) and PTL/PTL plus Ibu (182 lipid species), we found three common lipid species, diglyceride (4:0/20:5), phosphatidyl-ethanolamine (35:0p), and di-galactosyl-diacylglycerol (42:14), were downregulated (Figure 3E: Supplementary Table S4). These common upregulated and downregulated lipid species in the brains of Tulsi as well as Ibuprofen-treated mice suggested that both Tulsi and Ibuprofen might have similar effects on altering the lipidome of the ischemic brain. These results were also indicative that perhaps Tulsi acts as an anti-inflammatory agent similar to Ibuprofen. 

### 3.3. Effect of Tulsi in Modulating Lipidome Signature of Plasma in Mice with Photothrombotic Ischemic Lesion of the Cerebral Cortex

**Figure 4:**
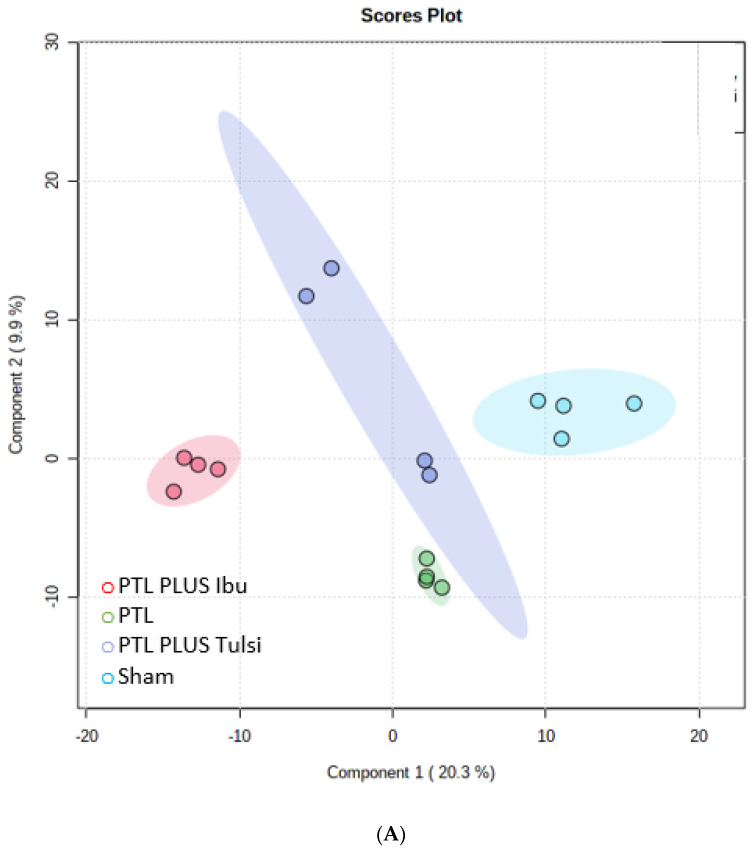

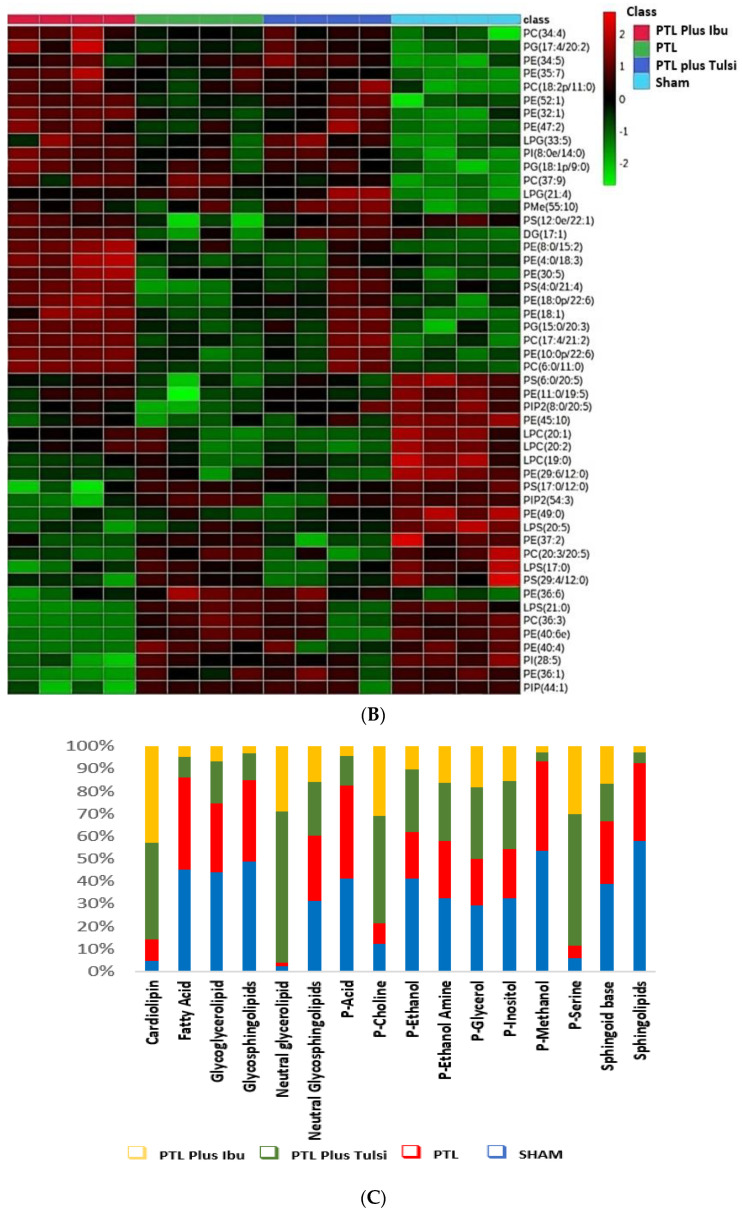

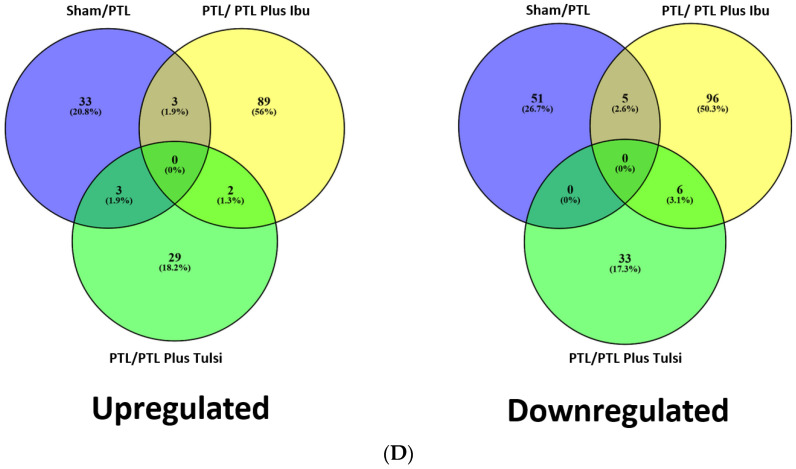
(**A**) PLS−DA score plot of lipid species in the plasma lipid samples of the sham, PTL, PTL plus Tulsi, and PTL plus Ibuprofen. (**B**) A heatmap showing the expression patterns of identified differential lipids in the plasma from different comparison groups. Upregulated lipids are shown in red while downregulated lipids are shown in green. (**C**) Relative abundance of different lipid groups in the plasma lipid samples of the sham, PTL, PTL plus Tulsi, and PTL plus Ibuprofen. (**D**) The common and unique upregulated and downregulated lipids were identified through the Venn diagram in the plasma samples of mice.

Untargeted lipidomic analysis was performed on plasma samples to identify markers of ischemic lesion and recovery, and other different types of comparisons were done on the plasma lipids as described for the brain (Figure 3A). PLS-DA analysis was performed to determine whether there were any significant differences in the plasma lipidomic profiles of different groups of mice. The results showed that there was significant variance in the plasma lipidomic profile of different comparison groups, as indicated by the component variances of PC1 (20.3%) and PC2 (9.9%) (Figure 4A).

This suggested that the different treatments significantly affected plasma lipid metabolism. Furthermore, the plasma lipidomics of the PTL plus Tulsi group was found to be similar to the sham group, which was consistent with the findings from the brain groups. This indicates that treatment with Tulsi was effective in restoring normal lipid metabolism not only in the brain but also in the plasma of lesioned animals. We employed hierarchical clustering analysis to explore the potential relationship between the lipids in the different groups. 

The heat map showed apparent differences in the top 50 lipid species (Figure 4B). The visual comparison suggests that lipid species in both PTL plus Tulsi and PTL plus Ibu have similar profiles. Volcano map of plasma samples (log_2_ FC= 1.5 and *p*-value < 0.05) of PTL group mice vs. sham group mice showed significant upregulation of 39 lipid molecules and downregulation of 56 lipid molecules (Supplementary Figure S11). Comparison of plasma lipids between PTL vs. PTL plus Tulsi using the volcano map analysis (log_2_ FC = 1.5 and *p* < 0.05) showed significant upregulation of 34 lipid species and downregulation of 39 lipid species (Supplementary Figure S14). While the volcano map analysis (log_2_ FC = 1.5 and *p* < 0.05) between PTL vs. PTL plus Ibu showed significant alterations in 201 lipid species (94 up- and 107 down-regulated; log_2_ FC = 1.5 and *p* < 0.05; Supplementary Figure S17). 

PLS-DA score plots for PTL vs. Sham (component 1 = 20.8%, component 2 = 18%; Supplementary Figure S12A), PTL vs. PTL plus Tulsi (component 1 = 27.4%, component 2 = 18.5%; Supplementary Figure S15A) and PTL vs. PTL plus Ibu (component 1 = 34.7%, component 2 = 17.7%; Supplementary Figure S18A) showed that these groups were distinct from each other. The VIP score of the relative concentration of lipid species in plasma of Sham vs. PTL (Supplementary Figure S12B), PTL vs. PTL plus Tulsi (Supplementary Figure S15B), and PTL vs. PTL plus Ibu (Supplementary Figure S18B) showed that several lipids were differentially regulated in the comparison groups. These differential alterations were further seen when heat map generated from hierarchical clustering analysis was examined between the PTL vs. Sham (Supplementary Figure S13A), PTL vs. PTL plus Tulsi (Supplementary Figure S16A), PTL vs. PTL plus Ibu (Supplementary Figure S19A). Using Random Forests analysis, the lipid species with significant alterations and ranked by the mean decrease in classification accuracy have been identified between PTL vs. Sham (Supplementary Figure S13B), PTL vs. PTL plus Tulsi (Supplementary Figure S16B), PTL vs. PTL plus Ibu (Supplementary Figure S19B). 

Relative abundance of lipids in the plasma of the Sham, PTL, PTL plus Tulsi, and PTL plus Ibu mice by arranging lipids into different groups (Figure 4C; Supplementary Table S2). Similar to the brain, a total of 16 lipid groups were found with high relative abundance in the plasma. Among the 16 groups, P-ethanol amine was highest in plasma samples of sham, PTL, PTL plus Tulsi, and PTL plus Ibu mice. P-inositol was also present in moderately high abundance in the plasma of all four groups of mice. 

The abundance of these two lipid groups was found to be highest In the sham group and reduced in the plasma of the PTL group. Interestingly, treatment with Tulsi was found to increase the abundance of P-ethanol amine and P-inositol groups, as well as neutral glycerolipids, P-serine, P-glycerol, and P-choline. Similarly, treatment with Ibuprofen also increased the abundance of these lipid groups, except P-glycerol. However, the relative abundance was found to be higher after treatment with Tulsi as compared to Ibuprofen. Furthermore, compared to the sham and lesion groups, there was a very high abundance of neutral glycerolipids, P-serine, cardiolipin, and P-choline in the plasma of Tulsi as well as Ibuprofen-treated mice. This suggested that treatment with Tulsi or Ibuprofen may have a significant impact on plasma lipid composition, which may have important implications for various physiological processes.

Venn analysis (Figure 4D) showed the upregulation of 39 lipid species and the downregulation of 56 lipid species in sham vs. PTL, and 95 upregulated and 107 downregulated lipid species in the plasma of PTL vs. PTL plus Ibu mice. In the plasma of PTL vs. PTL plus Tulsi, there were 34 upregulated and 39 downregulated lipid species (Figure 4D; Supplementary Tables S7 and S12). There are two common lipid species, PS (46:4) and PC (38:4), that are upregulated, and six common lipid species, PI (33:3/23:2), PE (44:11), PC (27:2), PE (4:0/19:5), PG (32:4), and PE (42:3), in the plasma of PTL vs. PTL plus Tusli and PTL vs. PTL plus Ibuprofen, which suggested that both Tulsi and Ibuprofen could be having a similar effect on these lipid species (Figure 4D; Supplementary Tables S5 and S6). 

### 3.4. Integration of Brain and Plasma Lipidomic Analysis 

**Figure 5:**
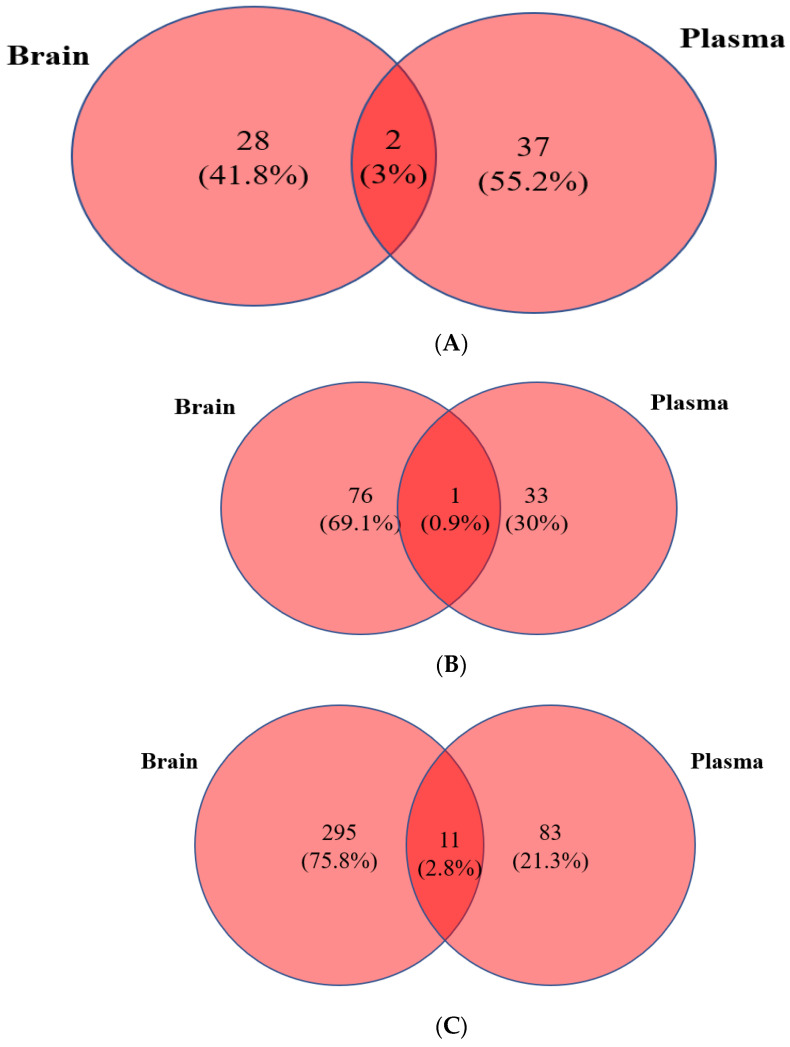
(**A**) Venny of upregulated lipid species in the brain and plasma between the Sham group and the PTL group. (**B**) Venny of upregulated lipid species in the brain and plasma between the PTL group and the PTL plus Tulsi group. (**C**) Venny of upregulated lipid species in the brain and plasma between the PTL group and the PTL plus Ibu group.

Although there were lipid species that were commonly altered in the plasma of both Tulsi- and Ibuprofen-treated animals, it may be possible that this could be indicative of a generalized reaction to either Tulsi or Ibuprofen. Hence, a comparative analysis of upregulated and downregulated lipid species in the brain and plasma of lesioned animals treated with Tulsi vs. Ibuprofen was performed. When sham vs. PTL conditions were compared between brain and plasma, there were 28 unique lipid species in the brain and 37 in the plasma, as well as two commonly upregulated lipid species, TG (8:0/24:6/24:7) and PS (8:0p/8:0) (Figure 5A; Supplementary Table S7). Analysis between PTL plus Tulsi and the PTL group showed only PE (10:0p/9:0) was commonly upregulated in the brain and plasma, though the brain and plasma showed 76 and 33 uniquely expressed lipid species, respectively (Figure 5B; Supplementary Table S9). A comparison of PTL plus Ibu and PTL only groups for the brain and plasma lipidome showed 11 lipid species commonly up-regulated. We also found 295 lipid species that were up-regulated and specific to the brain and 83 lipid species that were up-regulated and specific to the plasma (Figure 5C; Supplementary Table S11).

**Figure 6:**
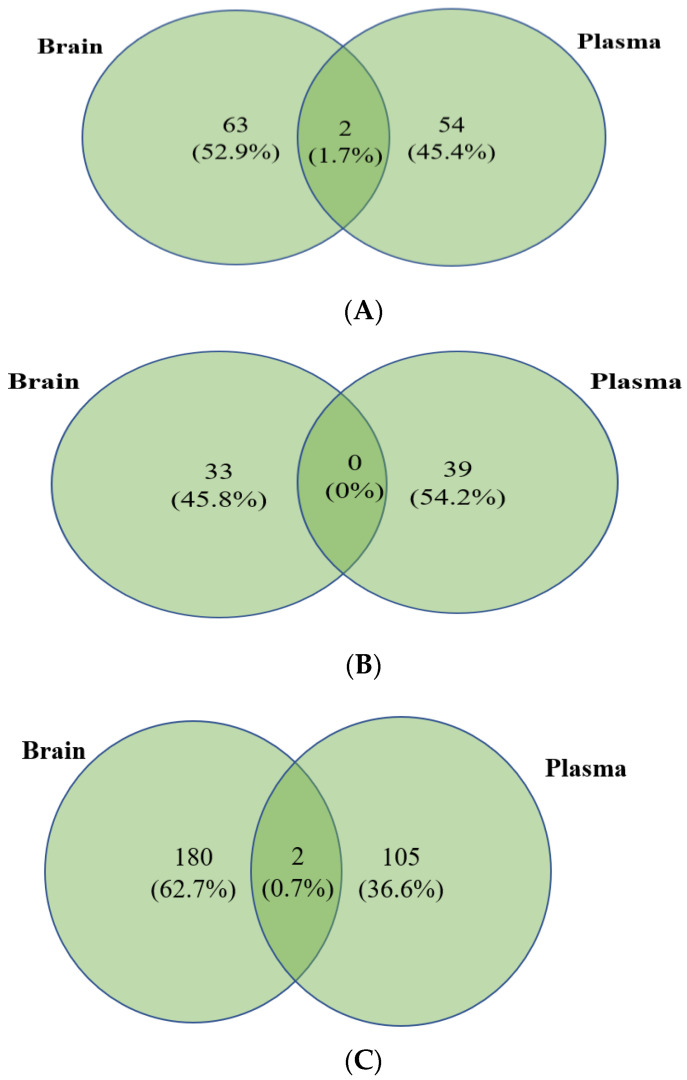
(**A**) Venny of downregulated lipid species in the brain and plasma between the Sham group and the PTL group. (**B**) Venny of downregulated lipid species of the brain and plasma between the PTL group and the PTL plus Tulsi group. (**C**) Venny of downregulated lipid species of the brain and plasma between the PTL group and the PTL plus Ibu group.

We have also analyzed the lipid species that were down-regulated in different groups. PTL as compared to sham showed significant down-regulation of lipid species: 63 in brains and 54 in plasma. The commonly downregulated lipid species were PE (45:10) and PE (42:3p) (Figure 6A, Supplementary Table S8). Interestingly, there were no commonly downregulated lipid species between PTL plus Tulsi and the PTL group. However, in lesioned animals treated with Tulsi, a significant reduction was seen for 33 lipid species in the brain and 39 lipid species in plasma (Figure 6B; Supplementary Table S10). 

Finally, a comparison of PTL plus Ibuprofen vs. PTL groups showed LPE (18:0) and PE (39:2) were commonly downregulated. A total of 180 lipid species were downregulated in the brain and 105 lipid species in plasma samples (Figure 6C; Supplementary Table S12). 

### 3.5. Untargeted Lipidomic and Metabolomics of Ocimum sanctum

**Figure 7:**
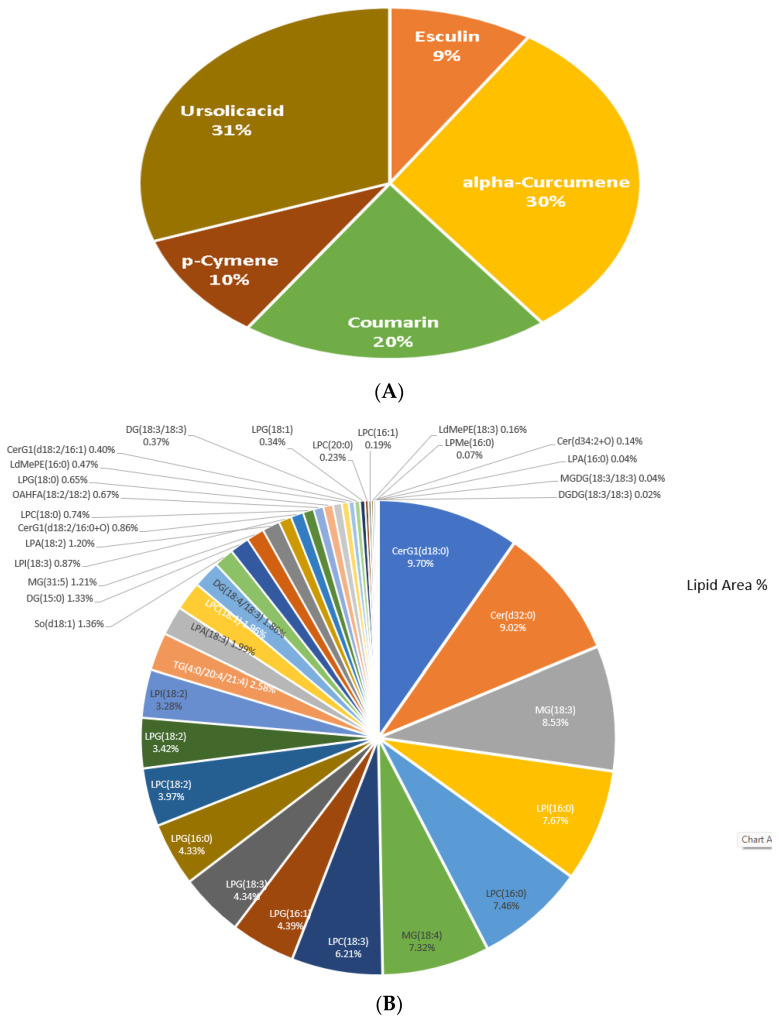

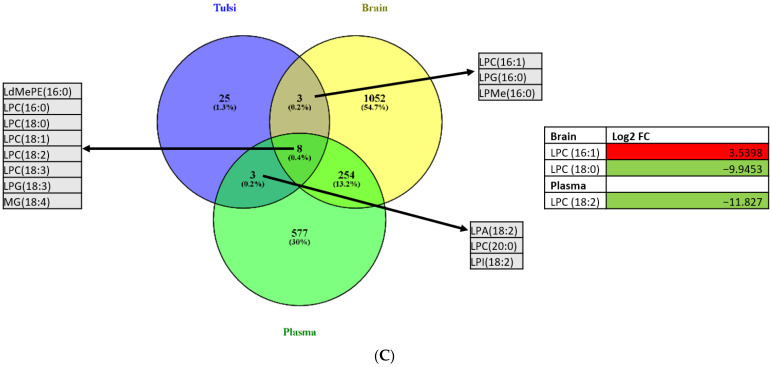
(**A**) Phytoconstituents in Tulsi extract. (**B**) A sunburst of different lipid molecules obtained in the Tulsi extract. (**C**) The common and unique lipids were identified through the Venn diagram in the Tulsi extract, brain cortex, and plasma samples of mice.

The extract of Tulsi leaves contains a diverse array of constituents that are known to possess potential biological activity. These bioactive compounds found in Tulsi extract are known for their various pharmacological properties and may contribute to the plant’s medicinal benefits. In the present study, we performed phytochemical screening of Tulsi extract. The standard protocol for phytochemical screening was employed to confirm the presence of metabolites and active compounds in the extract of Tulsi. Analysis of the ethanolic extract revealed the major presence of ursolic acid, alpha-curcumene, aesculin (esculin), coumarin, and p-cymene (Figure 7A), along with various other phytoconstituents in the extract of Tulsi, as listed in Supplementary Table S13.

Untargeted lipidomic analysis was also performed on the Tulsi extract to correlate the effect of Tulsi treatment on the lipidomic profiling of the brain and plasma after its treatment. We have obtained a total of 39 lipid molecules from the Tulsi extract (Figure 7B, Supplementary Table S14). 

Further, to find out lipids commonly found in tulsi extract, brain tissue, and plasma of the study group, we used a Venn diagram (Figure 7C) to find out lipid species that were modulated by Tulsi in the brain and plasma of mice from the PTL plus Tulsi group. Venn analysis showed eight lipid species were common with PTL plus the Tulsi group of the brain and plasma. These eight common lipid species were lyso-di-methyl phosphatidyl ethanolamine (16:0), lyso-phosphatidyl choline (16:0), lyso-phosphatidyl choline (18:0), lyso-phosphatidyl choline (18:1), lyso-phosphatidyl choline (18:2), lyso-phosphatidyl choline (18:3), lyso-phosphatidyl glycerol (18:3), monoglyceride (18:4). Venn analysis between lipid species of Tulsi and lipid species in the brain of PTL plus Tulsi has found three common lipids: lyso-phosphatidyl choline (16:1), lyso-phosphatidyl glycerol (16:0), and lyso-phosphatidyl methanol (16:0). Whereas Venn analysis of Tulsi lipids with plasma lipids of PTL plus Tulsi also found three common lipids: lyso-phosphatidic acid (18:2), lyso-phosphatidyl choline (20:0), and lyso-phosphatidyl inositol (18:2).

LPC (16:1) was found in Tulsi extract and found to be significantly increased in the brains of the PTL plus Tulsi-treated group. This finding provides valuable insights into the potential therapeutic effects of Tulsi extract in promoting healing and repair processes in the brain.

Our results show that lipidomic profiles in mouse models of ischemic injury in the brain and plasma were altered and distinct. These results also suggest that the alterations in some of the lipids seen with treatment with Tulsi or Ibuprofen were present in both the brain and plasma. These lipids might play a significant role in the recovery processes following ischemic injury. Importantly, the presence of these lipids in the circulatory system could help in the prognosis and evaluation of recovery. 

## 4. Discussion

Tulsi is well known for its neuroprotective action; in addition, the effect of ibuprofen on amelioration of brain lesions is also well known [45,46,47,48]. In the current pilot study, the lipidomic landscape of photothrombotic ischemic lesions in a mouse model was studied. We also examined the effect of the Tulsi treatment following a photothrombotic ischemic lesion on the modulation of the lipidomic profile of the brain and plasma in the mouse model. The alterations in lipidome profile with Tulsi treatment were compared to treatment with a non-steroid anti-inflammatory drug, Ibuprofen.

Our objective was to see whether changes in the brain and plasma lipidomic profiles could be attributed to the changes in the lesion status in the mouse model of ischemic stroke following treatment with Tulsi.

It has been shown in the past that oral administration of Tulsi to rats with MCAO (middle cerebral artery occlusion) markedly reduced the infarct size, reduced neurological deficits, and suppressed neuronal loss [31]. The potential of Tulsi in the management of experimentally induced cognitive dysfunctions in rats has been determined [49]. However, there was no data available about the effect of Tulsi on brain and plasma lipidomic profiles in ischemic stroke. This study represents the first untargeted lipidomic profiling of the brain and plasma to obtain information about the treatment effects of Tulsi after the manifestation of ischemia in the cerebral cortex of the brain.

Previous findings revealed that there is a strong relationship between lipid profiles and ischemic stroke [10]. Our comparison of sham and PTL mice in brain and plasma also showed a concordant effect with changes in the lipidomic profile, suggesting the effect of stroke upon the lipid species. We found the distinct lipidomic profile of the mouse model with the lesion, which, on treatment with either Tulsi or ibuprofen, showed apparent changes in the lipidomic profile that were systemic and evident even in the plasma samples. 

Ischemic brain injury releases free radicals in the form of hydroxyl radicals, radical superoxide anion, and nitric oxide (NO). During ischemic-stroke injury, R.O.S. are primarily produced in the mitochondria [50,51]. While excessive ROS formation secondary to reperfusion injury was attenuated by Tulsi [27]. Several active compounds present in Tulsi contribute to its anti-inflammatory activity [52,53,54,55,56]. Our study showed that after treatment with Tulsi, there was a change in the brain and plasma lipidomic profiles, which could possibly be one of the reasons for the decrease in lesion size seen post-treatment with Tulsi in previous studies [31]. 

Next, we classified the lipid species into groups to broadly understand the lipid groups involved in the treatment of photothrombotic ischemia with Tulsi. In the brain, lipid groups such as P-ethanol amine, P-choline, neutral glycerolipids, glycoglycerolipids, and others were significantly reduced in the lesion but surged after Tulsi treatment. On the other hand, in plasma, P-choline, neutral glycerolipids, P-serine, and P-glycerol were significantly increased compared to sham or lesion groups, which could be due to the systemic effect of Tulsi, which was administered orally to the mice. Increases in P-ethanol amine, P-choline, or P-glycerol were already known to have anti-inflammatory functions [57,58,59,60,61,62,63].

Further analysis between different comparison groups of the brain identified monoglactosyl diacylglycerol (MGDG) species that assist in the recovery process [64], which was found to be increased with the treatment of Tulsi or ibuprofen. The increased levels of MGDG (36:4) may be attributed to oral treatment with Tulsi or ibuprofen. Previous studies have found that MGDG has strong anti-inflammatory [65,66,67,68] and anti-proliferative activities [69]. It has been shown earlier that MGDG also has an inhibitory effect on cancer cells [70]. This was in concurrence with our results, which showed that the overall level of MGDG (36:4) was increased in the animals after the treatment with Tulsi and ibuprofen, though these observations require further study. 

Lipid species of phosphatidyl serine (PS), phosphatidyl ethanolamine (PE), lysyl-phosphatidylglycerol (LPG), and phosphatidyl inositol (PI) have shown common expression in sham and lesion plus Tulsi, which indicated the association of Tulsi with an increase in these lipids in the brain. These lipid groups were reported to perform several metabolic pathways that were found altered in disease conditions [71,72]. 

Further, we integrated the brain and plasma lipidomes in different comparison groups to attain site-specific or common alteration. The brain of Sham was found to be enriched explicitly in many species of glycerol lipids and to have reduced P-ethanol amine species, while in plasma, most of the altered lipid species belong to P-ethanol amine and P-serine compared to the lesion. These results were concordant with the literature [13,14,73,74]. The significant lipids that changed following treatment with Tulsi belong primarily to P-ethanolamine, while downregulated brain lipids were mainly composed of phosphatidyl serine. Phosphatidylserine is required for healthy nerve cell membranes, myelin, and cognitive functions [75,76]. Phosphatidylethanolamine was also found to be significantly altered in an ibuprofen-treated photothrombotic ischemia animal model. All this suggests that changes in phosphatidyl ethanolamine and phosphatidyl serine were induced due to treatment with Tulsi or ibuprofen and may be associated with lesion regression. It can also be considered for systemic representation of site-specific injury or improvement. 

Metabolomic analysis of Tulsi leaf extract has provided robust evidence confirming the presence of several important phytoconstituents. Notably, ursolic acid, alpha-curcumene, aesculin (Esculin), coumarin, and p-Cymene have been identified as significant components in the Tulsi extract, as supported by existing literature. [77,78]. The identification and confirmation of these phytoconstituents, alongside other metabolites in Tulsi extract through metabolomic analysis, provide scientific support for the traditional uses and health-promoting effects associated with Tulsi. These findings expand our understanding of the chemical composition of Tulsi and pave the way for further exploration of its therapeutic applications. It was reported in the literature that the leaf extract of Tulsi contains a higher concentration of ursolic acid [79]. Ursolic acid has several medicinal properties such as analgesic, anti-inflammatory, anti-atherosclerotic, anti-cancer, anti-diabetic, anti-epileptic, hepato-protective, anti-hyperlipidemic, anti-fertility, anti-platelet aggregation, anti-tuberculosis, and anti-HIV activities [80,81,82,83,84,85]. Ursolic acid, derived from *Ocimum sanctum*, has been recognized for its potent anti-inflammatory properties. The administration of Ursolic acid has been shown to reduce brain edema and neurological insufficiencies after TBI induction in a murine model [86]. Ursolic acid has significantly helped in reducing intercellular adhesion molecule-1 (ICAM-1), toll-like receptor 4 (TLR4), nuclear factor-*κ*B (NF-*κ*B) P65, interleukin-1*β* (IL-1*β*), tumor necrosis factor-*α* (TNF-*α*), interleukin-6 (IL-6), inducible nitric oxide synthase (iNOS), and matrix metalloproteinase-(MMP)-9 in a subarachnoid hemorrhage brain injury model in rats [87,88]. In the MCAO model in rats, administration of ursolic acid helped decrease neurological deficits along with reduced levels of proinflammatory cytokine concentrations (IL-1*β*, TNF-*α*, and IL-6), TLR4, and inactivated NF-*κ*B [89]. It also suppressed the activity of cyclooxygenase-2 (COX-2), an enzyme involved in the synthesis of inflammatory prostaglandins [89]. Ursolic acid, being the active constituent of Tulsi, may be responsible for its anti-inflammatory action, as numerous studies have highlighted the anti-inflammatory effects of ursolic acid, making it a promising therapeutic agent for various inflammatory conditions.

Untargeted lipidomic analysis of the Tulsi extract has shown the presence of 39 important lipid molecules, which may help in regulating the perturbed lipidomic of the brain and plasma and might be responsible for the recovery process in various disease ailments. LPC (16:1), an important lipid in Tulsi extract, was found to be upregulated in the brains of PTL after treatment with Tulsi. Lysophosphatidylcholine (LPC) was considered an important membrane constituent implicated in signaling and immune regulation [90]. It was reported in the literature that the level of LPC was altered in the brain following both focal and global cerebral ischemia in rats and mice [13,91,92,93,94]. LPC is secreted from apoptotic cells, which play a role in the inflammatory reaction mediated by microglia [95]. The observed elevation of LPC (16:1) suggested a potential involvement of Tulsi lipids in the recovery process following brain lesions. Therefore, targeting LPC (16:1) might be a potential therapeutic method for brain ischemia. However, further studies are needed to fully elucidate the mechanisms underlying the role of Tulsi lipids, including LPC (16:1), in brain recovery. The current pilot study aimed to generate preliminary data and valuable insights that would guide future research. The findings from this study provided valuable insights into the potential therapeutic effects of Tulsi extract in promoting healing and repair processes in the brain and plasma. However, considering the intricate nature of stroke and its multifaceted pathophysiology, it is crucial to go beyond the identification of lipidomic changes. Establishing a clear correlation between lipidomic changes and the overall improvement or deterioration of stroke outcomes is essential. Hence, a future larger-scale study with a more substantial sample size along with examining and assessing the correlation between inflammatory markers, neurological function, infarct size measurement, functional recovery, anti-inflammatory markers, neurotransmitters, and the mechanisms of action of treatments should be planned to gain comprehensive insights into stroke management and treatment efficacy.

## 5. Conclusions

Nowadays, lipidomic-based studies have become an important tool for obtaining lipidomic snapshots. In the present study, we have performed metabolomics and untargeted lipidomic analysis of the Tulsi extract to see the presence of lipid molecules and various other metabolites in the extract and further correlated the effect of treatment of the Tulsi extract after a photothrombotic lesion on the modulation of lipidomic profiling in the brain and plasma. This approach will provide insights into the roles of specific lipids and help establish a therapeutic solution for human stroke and related disorders. Our study found the deregulation of various lipid species as a characteristic feature of the mouse model of an ischemic stroke lesion. We also reported that brain and plasma lipids were altered in animals with stroke-like lesions following treatment with Tulsi extract. Specifically, lipid species such as Phosphatidyl Serine (PS), Phosphatidyl Ethanolamine (PE), Lysyl-phosphatidylglycerol (LPG), and Phosphatidyl Inositol (PI) were increased after treatment with Tulsi. Notably, the cortex of mice treated with Tulsi showed an upregulation of Monogalactosyldiacylglycerol (36:4). One intriguing finding was the significant increase of LPC (16:1), present in Tulsi extract, in the brains of the PTL plus Tulsi-treated group. Our study suggested that significant changes in the lipidomic profile in the brain and plasma caused by either Tulsi or Ibuprofen may help ameliorate brain injury. The change in the brain and plasma lipidomic profile induced by Tulsi could be relevant to the reduction of lesion seen after Tulsi treatment in previous stroke studies; however, further validation with various stroke-related measures is needed to draw more robust conclusions. 

## Data Availability

Data are contained within the article.

## Supplementary Materials

The following supporting information can be downloaded at: https://www.mdpi.com/article/10.3390/life13091877/s1, Figure S1: Random Forest from mice brains of different groups identified 15 different lipid features and ranked by the mean decrease in classification accuracy. Figure S2: The volcano map showed the differential lipids expression level in the mice brain of sham vs PTL groups (log2 FC = 1.5 and *p*-value < 0.05 considered significant). Upregulated lipids are shown by red color dots while downregulated lipids are shown by blue colored dots. Figure S3: (A): Partial Least Squares-Discriminant Analysis (PLS-DA) plot of lipid species in mice brain of sham vs PTL groups revealing the component variance 19.2% and 17.8%. (B): VIP score of lipid species in PLS-DA in the mice brain of sham vs. PTL groups. The colored boxes on the right side represent the relative concentration of the corresponding lipid species. Red indicates high and blue indicates low. Figure S4: (A): Heatmap of the expression of identified differential lipids in the mice brain between sham vs PTL groups. The colored boxes on the right side represent the relative concentration of the corresponding lipid species. Red indicates high and green indicates low. (B): Random Forest from mice brains of sham vs PTL groups identified 15 different lipid features and ranked by the mean decrease in classification accuracy. Figure S5: The volcano map showed the differential lipids expression level in the mice brain of PTL vs. PTL plus Tulsi groups (log2 FC = 1.5 and *p*-value < 0.05 considered significant). Upregulated lipids are shown by red color dots while downregulated lipids are shown by blue colored dots. Figure S6: (A): Partial Least Squares - Discriminant Analysis (PLS-DA) plot of lipid species in mice brain of PTL vs. PTL plus Tulsi groups revealing the component variance 36.1% and 14.3%. (B): VIP score of lipid species in PLS-DA in the mice brain of PTL vs. PTL plus Tulsi groups. The colored boxes on the right side represent the relative concentration of the corresponding lipid species. Red indicates high and blue color indicates low. Figure S7: (A): Heatmap of the expression of identified differential lipids in the mice brain between PTL vs. PTL plus Tulsi groups. The colored boxes on the right side represent the relative concentration of the corresponding lipid species. Red indicates high and green indicates low. (B): Random Forest from mice brains of PTL vs. PTL plus Tulsi groups identified 15 different lipid features and ranked by the mean decrease in classification accuracy. Figure S8: The volcano map showed the differential lipids expression level in the mice brain of PTL vs. PTL plus Ibu groups (log2 FC = 1.5 and *p*-value < 0.05 considered significant). Upregulated lipids are shown by red color dots while downregulated lipids are shown by blue colored dots. Figure S9: (A): Partial Least Squares - Discriminant Analysis (PLS-DA) plot of lipid species in mice brain of PTL vs. PTL plus Ibu groups revealing the component variance 47.1% and 12.5%. (B): VIP score of lipid species in PLS-DA in the mice brain of PTL vs. PTL plus Ibu groups. The colored boxes on the right side represent the relative concentration of the corresponding lipid species. Red color indicates high and blue indicates low. Figure S10: (A): Heatmap of the expression of identified differential lipids in the mice brain between PTL and PTL plus Ibu groups. The colored boxes on the right side represent the relative concentration of the corresponding lipid species. Red indicates high and green indicates low. (B): Random Forest from mice brains of PTL vs. PTL plus Ibu groups identified 15 different lipid features and ranked by the mean decrease in classification accuracy. Figure S11: The volcano map showed the differential lipids expression level in the mice plasma of sham vs PTL groups (log2 FC = 1.5 and *p*-value < 0.05 considered significant). Upregulated lipids are shown by red color dots while downregulated lipids are shown by blue colored dots. Figure S12: (A): Partial Least Squares - Discriminant Analysis (PLS-DA) plot of lipid species in mice plasma of sham vs. PTL groups revealing the component variance 20.8% and 18%. (B): VIP score of lipid species in PLS-DA in the mice plasma of sham vs PTL groups. The colored boxes on the right side represent the relative concentration of the corresponding lipid species. Red color indicates high and blue color indicates low. Figure S13: (A): Heatmap of the expression of identified differential lipids in the mice plasma between sham vs PTL groups. The colored boxes on the right side represent the relative concentration of the corresponding lipid species. Red indicates high and green indicates low. (B): Random Forest from mice plasma of sham vs. PTL groups identified 15 different lipid features and ranked by the mean decrease in classification accuracy. Figure S14: The volcano map showed the differential lipids expression level in the mice plasma of PTL vs PTL plus Tulsi groups (log2 FC = 1.5 and *p*-value < 0.05 considered significant). Upregulated lipids are shown by red color dots while downregulated lipids are shown by blue colored dots. Figure S15: (A): Partial Least Squares-Discriminant Analysis (PLS-DA) plot of lipid species in mice plasma of PTL vs PTL plus Tulsi groups revealing the component variance 27.4% and 18.5%. (B): VIP score of lipid species in PLS-DA in the mice plasma of PTL vs PTL plus Tulsi groups. The colored boxes on the right side represent the relative concentration of the corresponding lipid species. Red indicates high and blue indicates low. Figure S16: (A): Heatmap of the expression of identified differential lipids in the mice plasma between PTL vs. PTL plus Tulsi groups. The colored boxes on the right side represent the relative concentration of the corresponding lipid species. Red indicates high and green indicates low. (B): Random Forest from mice plasma of PTL vs. PTL plus Tulsi groups identified 15 different lipid features and ranked by the mean decrease in classification accuracy. Figure S17: The volcano map showed the differential lipids expression level in the mice plasma of PTL vs PTL plus Ibu groups (log2 FC = 1.5 and *p*-value < 0.05 considered significant). Upregulated lipids are shown by red color dots while downregulated lipids are shown by blue colored dots. Figure S18: (A): Partial Least Squares-Discriminant Analysis (PLS-DA) plot of lipid species in mice plasma of PTL vs. PTL plus Ibu groups revealing the component variance 34.7% and 17.7%. (B): VIP score of lipid species in PLS-DA in the mice plasma of PTL vs. PTL plus Ibu groups. The colored boxes on the right side represent the relative concentration of the corresponding lipid species. Red indicates high and blue indicates low. Figure S19: (A): Heatmap of the expression of identified differential lipids in the mice plasma between PTL vs. PTL plus Ibu groups. The colored boxes on the right side represent the relative concentration of the corresponding lipid species. Red indicates high and green indicates low. (B): Random Forest from mice plasma of PTL vs. PTL plus Ibu groups identified 15 different lipid features and ranked by the mean decrease in classification accuracy. Table S1: Table showing relative abundance of lipid groups in the mice brain among the Sham group, PTL group, PTL plus Tulsi group and PTL plus Ibu groups. Table S2: Table showing relative abundance of lipid groups in the mice plasma among the Sham group, PTL group, PTL plus Tulsi group and PTL plus Ibu groups. Table S3: Venny brain upregulated lipid species among the Sham group, PTL group, PTL plus Tulsi group and PTL plus Ibu groups. Table S4: Venny brain downregulated lipid species among the Sham group, PTL group, PTL plus Tulsi group and PTL plus Ibu groups. Table S5: Venny plasma upregulated lipid species among the Sham group, PTL group, PTL plus Tulsi group and PTL plus Ibu groups. Table S6: Venny plasma downregulated lipid species among the Sham group, PTL group, PTL plus Tulsi group and PTL plus Ibu groups. Table S7: Venny of upregulated lipid species of brain and plasma between the Sham group and PTL group. Table S8: Venny of downregulated lipid species of brain and plasma between the Sham group and PTL group. Table S9: Venny of upregulated lipid species of brain and plasma between PTL group and PTL plus Tulsi group. Table S10: Venny of downregulated lipid species of brain and plasma between PTL group and PTL plus Tulsi group. Table S11: Venny of upregulated lipid species of brain and plasma between PTL group and PTL plus Ibuprofen group. Table S12: Venny of downregulated lipid species of brain and plasma between PTL group and PTL plus Ibuprofen group. Table S13: Phytoconstituents in the Tulsi extract. Table S14: List of Lipid molecules obtained from Tulsi extract. Supplementary document File S2.

## Abbreviations

ANOVA—Analysis of Variance, FWHM—Full Width at Half Maximum, HCD—Higher-Energy Collisional Dissociation, HESI—Heated Electrospray Ionization, IV—Intravenous, LdMePE—lyso-dimethylphosphatidylethanolamine, LPA—lysophosphatidic acid, LPC—Lysyl-Phosphatidyl Choline, LPE—lysophosphatidylethanolamine, LPG—Lysyl-phosphatidylglycerol, LPI—lysophosphatidylinositol, LPMe—lysophosphatidylmethanol, MCAO—Middle Cerebral Artery Occlusion, MG—monoglyceride, MGDG—Monogalactosyldiacylglycerol, PCA—Principal Component Analysis, PC—Phosphatidyl Choline, PE—Phosphatidyl Ethanolamine, P-Glycerol—Phosphatidyl Glycerol, PI—Phosphatidyl Inositol, PLS-DA—Partial Least Squares-Discriminant Analysis, PS—Phosphatidyl Serine, PTL—Photothrombotic Lesion.
