# Supplementary material for: Ocimum sanctum Alters the Lipid Landscape of the Brain Cortex and Plasma to Ameliorate the Effect of Photothrombotic Stroke in a Mouse Model
Source: Life (Basel). 2023 Sep 7;13(9):1877. doi: 10.3390/life13091877 (PMC10533110; doi:10.3390/life13091877)
Supplement: Supplementary file 1 [file life-13-01877-s001.zip › Supplementary Document file S2.pdf]

Lipid Search software parameters:

Search type: Product ion search

Exp type: LC-MS

Precursor tolerance: ~5–7 ppm

Product tolerance: 7–10 ppm

Intensity threshold: 1.00%

height-Score threshold: 8

Execute Quantitation: On

M/z tolerance (quantitation): −/plus 5.0 ppm

RT range (min.) (quantitation): −/plus 0.5

Top rank filter: On

Main node filter: Main isomer peak

m-Score threshold: 5

c-Score threshold: 2

FA priority: On

ID Quality Filter: Check A, B, and C or A–D for lower quality IDs

Target class: Select ALL lipid classes

Ion adducts (pos): plusH, plusNH_4 and plus2 H

Ion adducts (neg): −H, plusHCOO and −2H

Lipid Extraction method: -

Briefly, 100µL brain/plasma samples were added to chloroform: methanol (2:1) mixture and homogenized. Thereafter, the samples were centrifuged at 13,000rpm for 10 minutes to extract the dissolved lipids (supernatant). Each sample was then vacuum dried. These dried samples were reconstituted in 100 µL of 65:30:5:5 standard solution consisting of acetonitrile (65%): isopropanol (30%): water (5%): internal and external standards (5%) and were then subjected to reverse-phase chromatography in C18 column (Thermo Scientific™25003102130: 3 µm,

2.1 mm, 100 mm) using ultra-high-performance liquid chromatographic system. Mobile phase A was 0.1 % formic acid and mobile phase B was 100% acetonitrile.

Mass spectrometry procedure:

The HESI source parameters were as follows: the spray voltage was set to 3.7 kV in positive and −3.1 kV in negative ionization mode. The heated capillary temperature was maintained at 360 °C and the sheath and auxiliary gas flow were set to 15 and 10 (arbitrary units), respectively. All the samples were pooled together and spiked with internal standards (Dinose b: 1mg/mL; MCPA: 1mg/mL; Dimetrazole: 1mg/mL) and external standards (Cholesterol: 0.1mg/mL; Colchicine: 4mg/mL; Impramine: 2mg/mL; Roxithromycin: 2mg/mL; Amiloride: 1mg/mL; Atropine:2mg/mL; 2-aminoanthroceae: 370.5mg/mL; Prednisolone: 2mg/mL) to make dilutions of 1:1,1:2,1:4 and 1:8 which were operated as QC for higher-energy collisional dissociation (HCD) MS/MS experiment. We in this study used metabolite internal and external standard to attain analytical sensitivity for the MS/MS experiments. In MS/MS mode isolation width was set to *m/z* 1.5, the normalized collision energy was 32 % and the mass resolution was set at 17,500 FWHM at *m/z* 200.
