# Supplementary material for: Ocimum sanctum Alters the Lipid Landscape of the Brain Cortex and Plasma to Ameliorate the Effect of Photothrombotic Stroke in a Mouse Model
Source: Life (Basel). 2023 Sep 7;13(9):1877. doi: 10.3390/life13091877 (PMC10533110; doi:10.3390/life13091877)
Supplement: Supplementary file 1 [file life-13-01877-s001.zip › Supplementary figures file S1.pdf]

Supplementary Figure S1: Random Forest from mice brains of different groups identified 15 different lipid features and ranked by the mean decrease in classification accuracy.

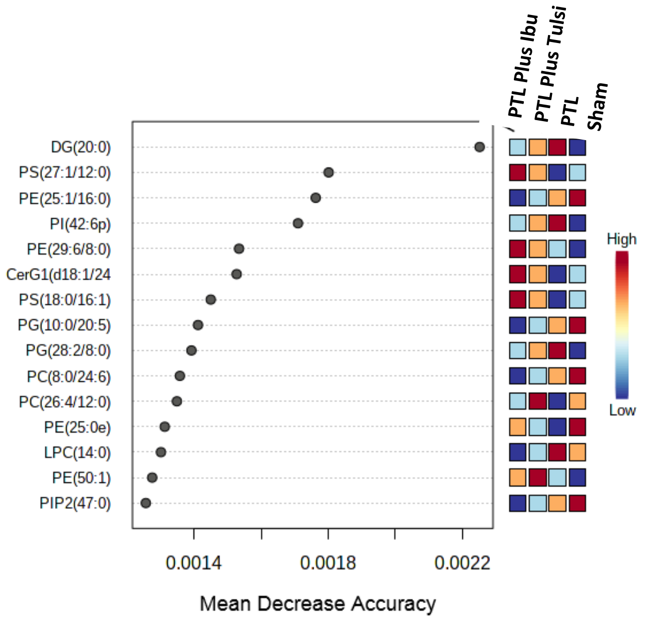

Supplementary Figure S2: The volcano map showed the differential lipids expression level in the mice brain of sham vs PTL groups (log2 FC= 1.5 and p-value <0.05 considered significant). Upregulated lipids are shown by red colour dots while downregulated lipids are shown by blue coloured dots.

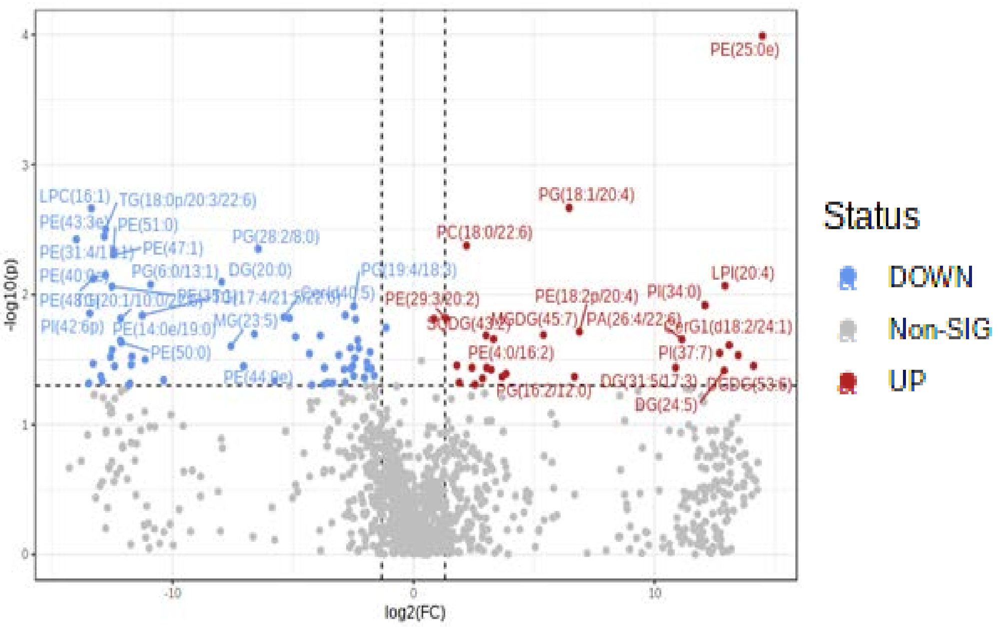

Supplementary Figure S3: (A) Partial Least Squares - Discriminant Analysis (PLS-DA) plot of lipid species in mice brain of sham vs PTL groups revealing the component variance 19.2% and 17.8%. **(B)** VIP score of lipid species in PLS-DA in the mice brain of sham vs PTL groups. The coloured boxes on the right side represent the relative concentration of the corresponding lipid species. Red color indicates high and blue color indicates low.

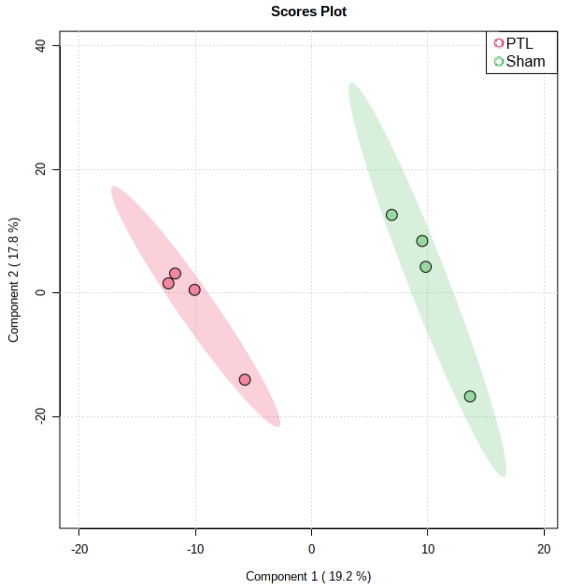

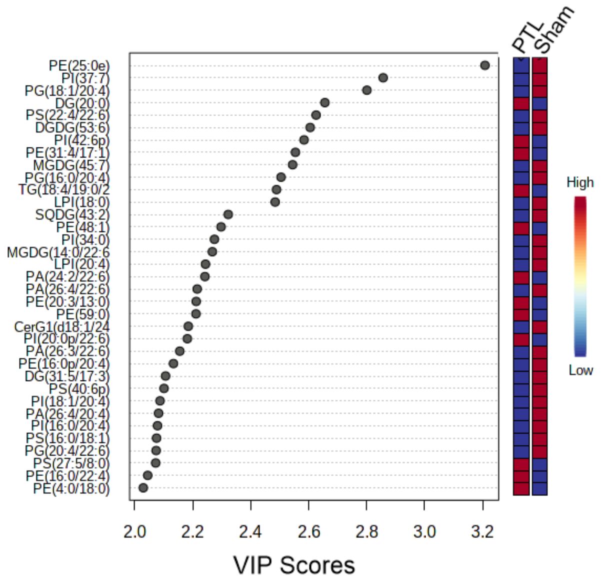

(A) **(B)**

Supplementary Figure S4: (A) Heatmap of the expression of identified differential lipids in the mice brain between sham vs PTL groups. The coloured boxes on the right side represent the relative concentration of the corresponding lipid species. Red color indicates high and green color indicates low. **(B)** Random Forest from mice brains of sham vs PTL groups identified 15 different lipid features and ranked by the mean decrease in classification accuracy.

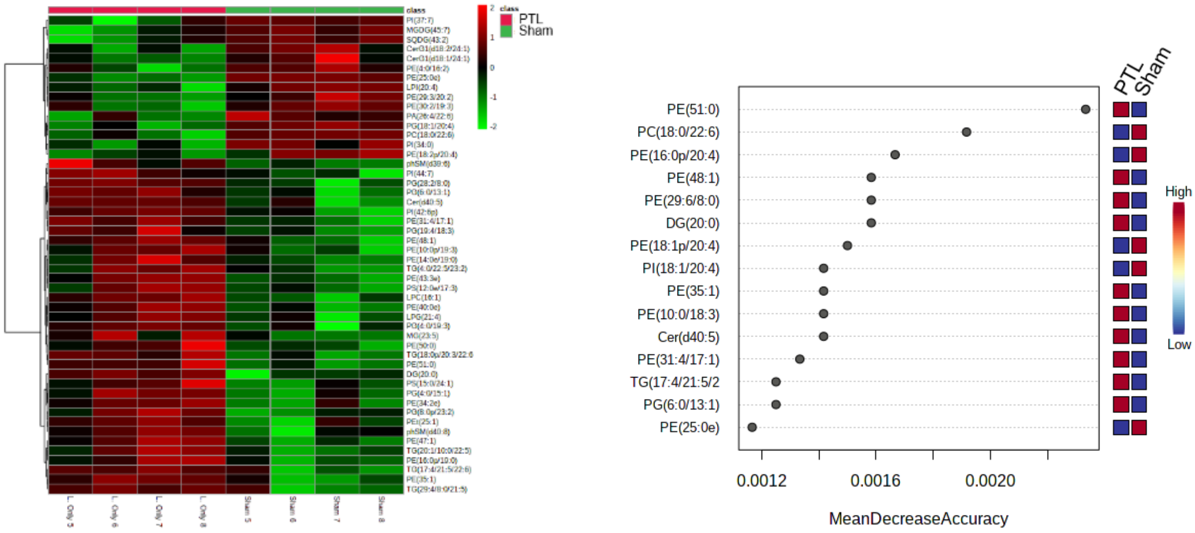

(A) **(B)**

Supplementary Figure S5: The volcano map showed the differential lipids expression level in the mice brain of PTL vs PTL plus Tulsi groups (log2 FC= 1.5 and p-value <0.05 considered significant). Upregulated lipids are shown by red colour dots while downregulated lipids are shown by blue coloured dots.

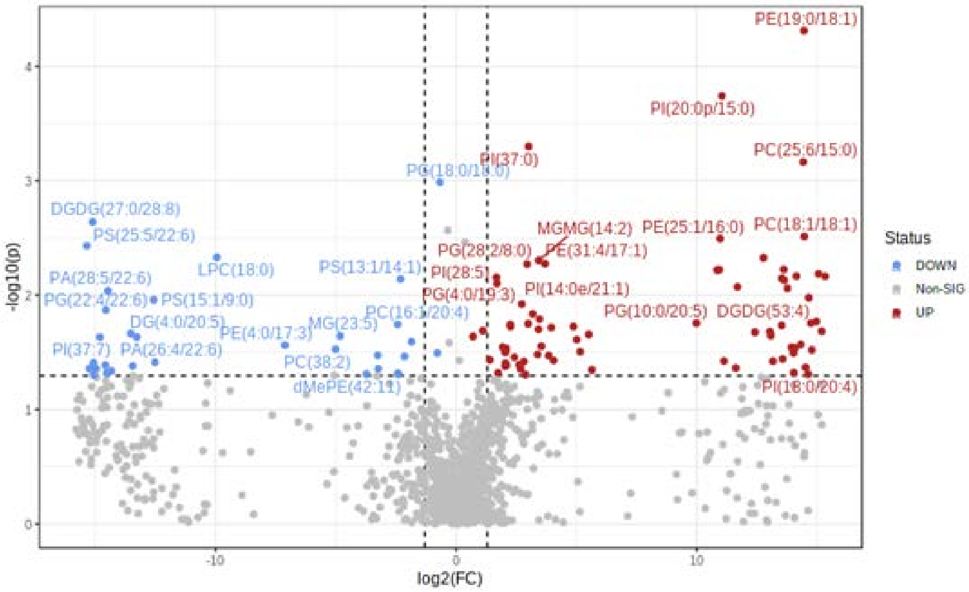

Supplementary Figure S6: **(A)** Partial Least Squares - Discriminant Analysis (PLS-DA) plot of lipid species in mice brain of PTL vs PTL plus Tulsi groups revealing the component variance 36.1% and 14.3%. **(B)** VIP score of lipid species in PLS-DA in the mice brain of PTL vs PTL plus Tulsi groups. The coloured boxes on the right side represent the relative

concentration of the corresponding lipid species. Red color indicates high and blue color indicates low.

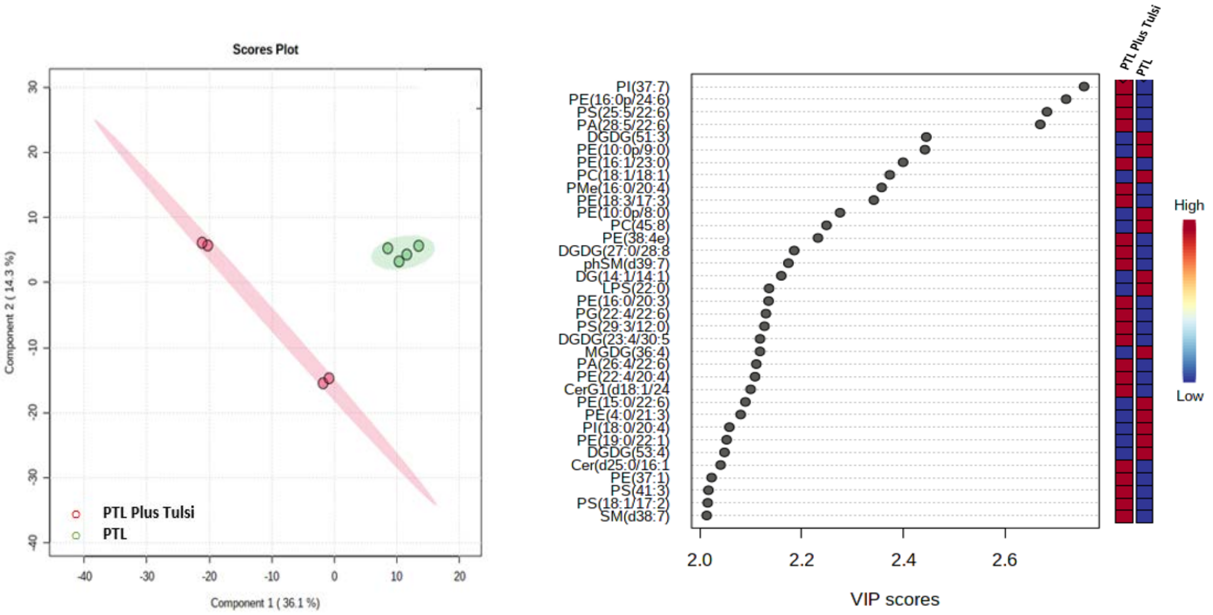

(A) **(B)**

Supplementary Figure S7: (A) Heatmap of the expression of identified differential lipids in the mice brain between PTL vs PTL plus Tulsi groups. The coloured boxes on the right side represent the relative concentration of the corresponding lipid species. Red color indicates high and green color indicates low. **(B)** Random Forest from mice brains of PTL vs PTL plus Tulsi groups identified 15 different lipid features and ranked by the mean decrease in classification accuracy.

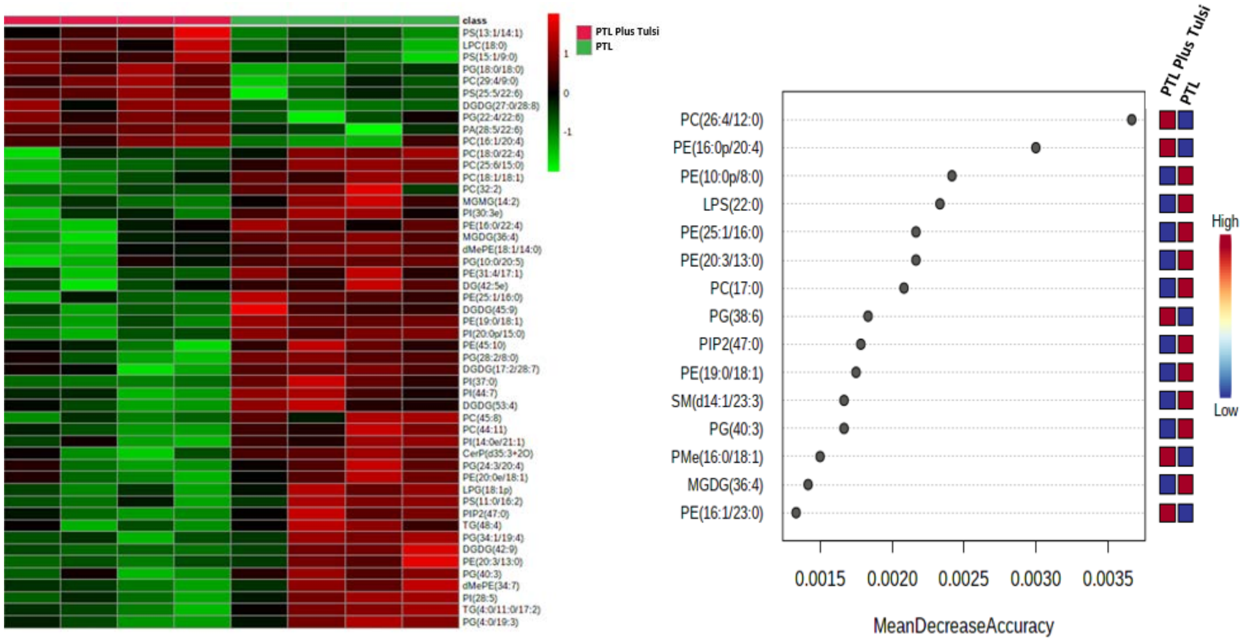

(A) (B)

Supplementary Figure S8: The volcano map showed the differential lipids expression level in the mice brain of PTL vs PTL plus Ibu groups (log2 FC= 1.5 and p-value <0.05 considered significant). Upregulated lipids are shown by red colour dots while downregulated lipids are shown by blue coloured dots.

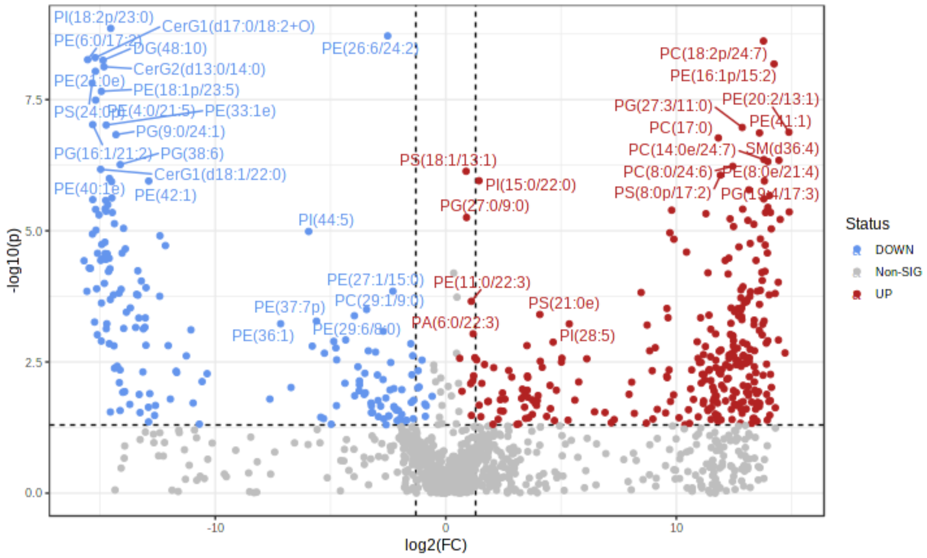

Supplementary Figure S9: (A) Partial Least Squares - Discriminant Analysis (PLS-DA) plot of lipid species in mice brain of PTL vs PTL plus Ibu groups revealing the component variance 47.1% and 12.5%. **(B)** VIP score of lipid species in PLS-DA in the mice brain of PTL vs PTL plus Ibu groups. The coloured boxes on the right side represent the relative concentration of the corresponding lipid species. Red color indicates high and blue color indicates low.

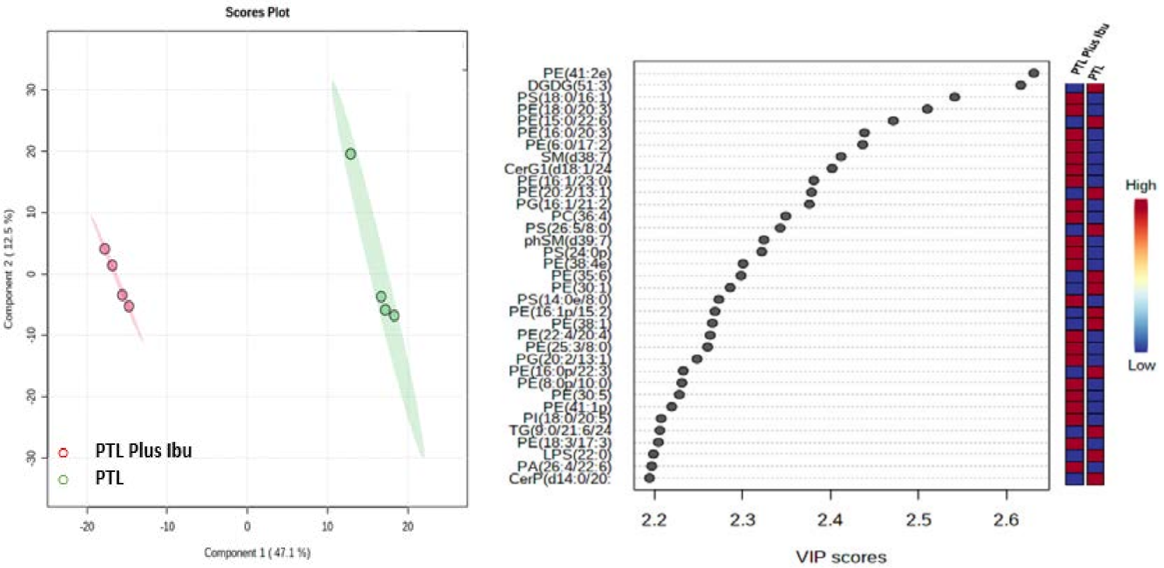

(A) (B)

Supplementary Figure S10: (A) Heatmap of the expression of identified differential lipids in the mice brain between PTL vs PTL plus Ibu groups. The coloured boxes on the right side represent the relative concentration of the corresponding lipid species. Red color indicates high and green color indicates low. **(B)** Random Forest from mice brains of PTL vs PTL plus Ibu groups identified 15 different lipid features and ranked by the mean decrease in classification accuracy.

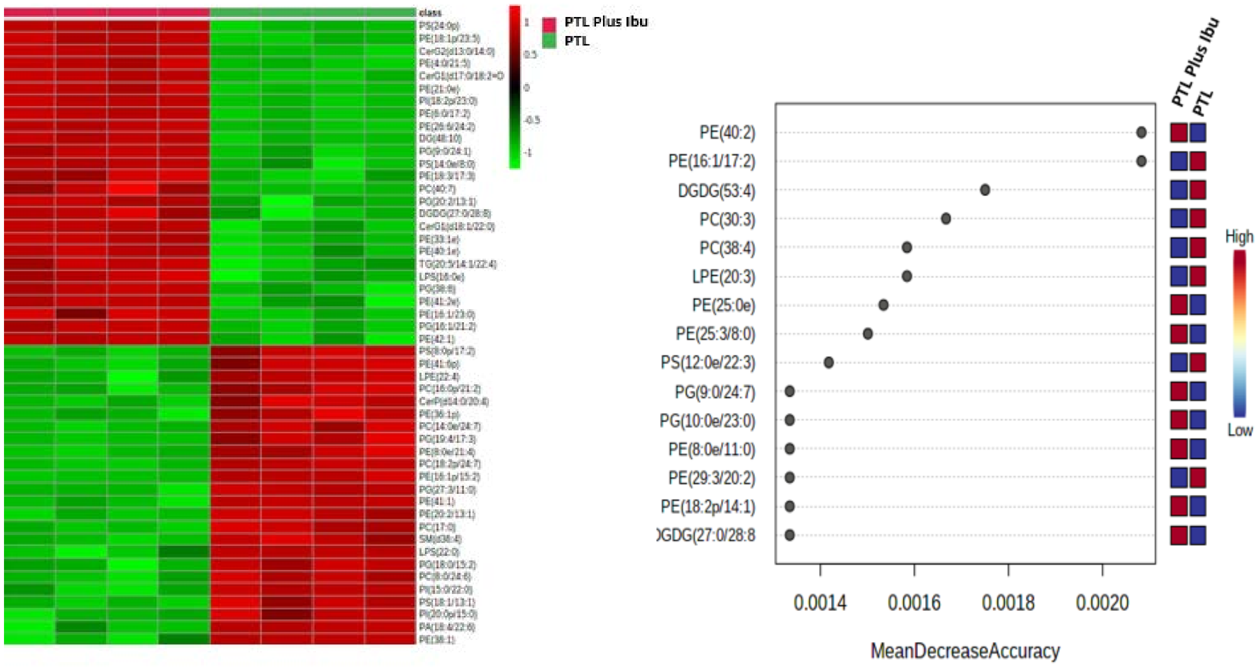

(A) **(B)**

Supplementary Figure S11: The volcano map showed the differential lipids expression level in the mice plasma of sham vs PTL groups (log2 FC= 1.5 and p-value <0.05 considered significant). Upregulated lipids are shown by red colour dots while downregulated lipids are shown by blue coloured dots.

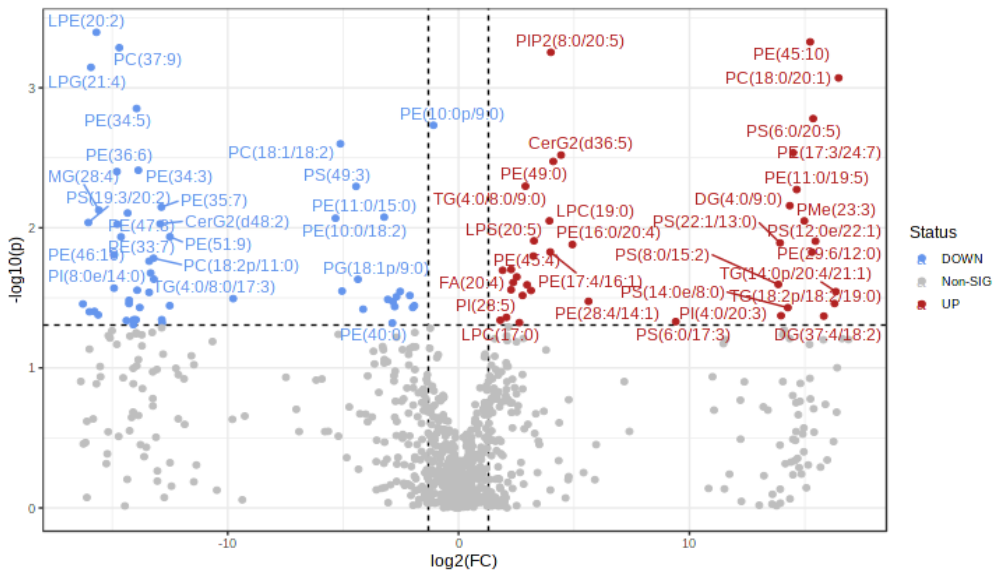

Supplementary Figure S12: (A) Partial Least Squares - Discriminant Analysis (PLS-DA) plot of lipid species in mice plasma of sham vs PTL groups revealing the component variance 20.8% and 18%. **(B)** VIP score of lipid species in PLS-DA in the mice plasma of sham vs PTL groups. The coloured boxes on the right side represent the relative concentration of the corresponding lipid species. Red color indicates high and blue color indicates low.

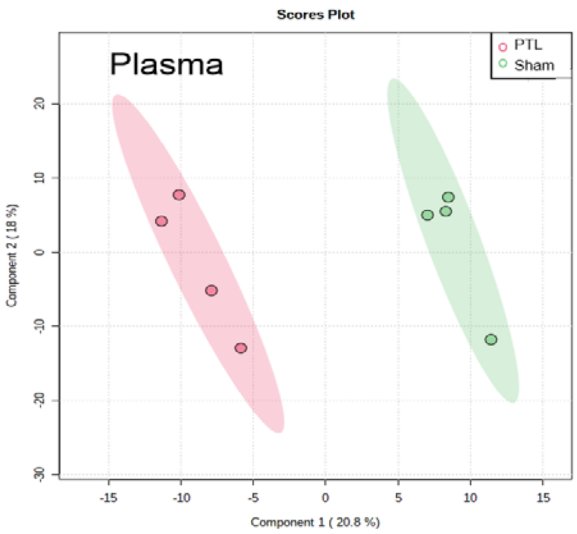

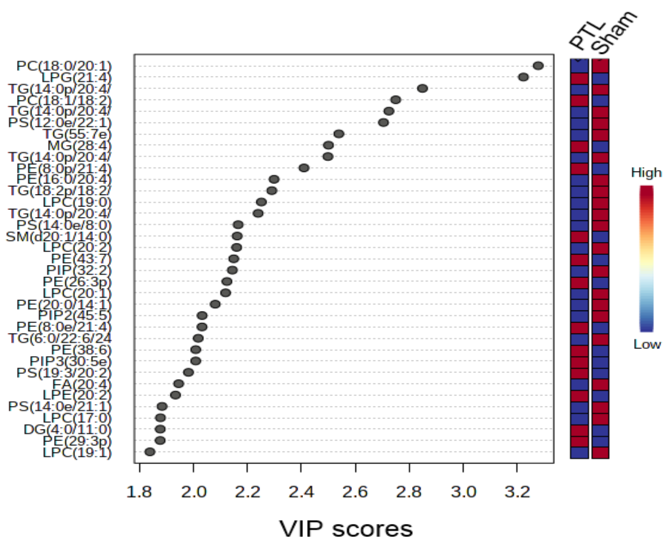

(A) **(B)**

Supplementary Figure S13: (A) Heatmap of the expression of identified differential lipids in the mice plasma between sham vs PTL groups. The coloured boxes on the right side represent the relative concentration of the corresponding lipid species. Red color indicates high and green color indicates low. **(B)** Random Forest from mice plasma of sham vs PTL groups identified 15 different lipid features and ranked by the mean decrease in classification accuracy.

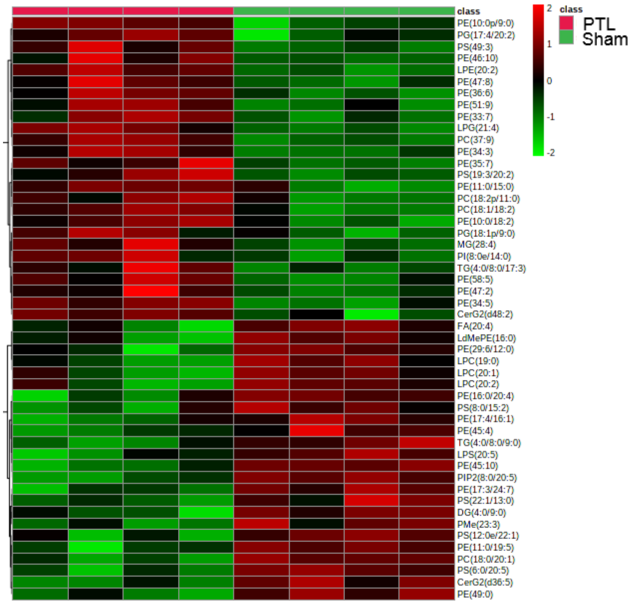

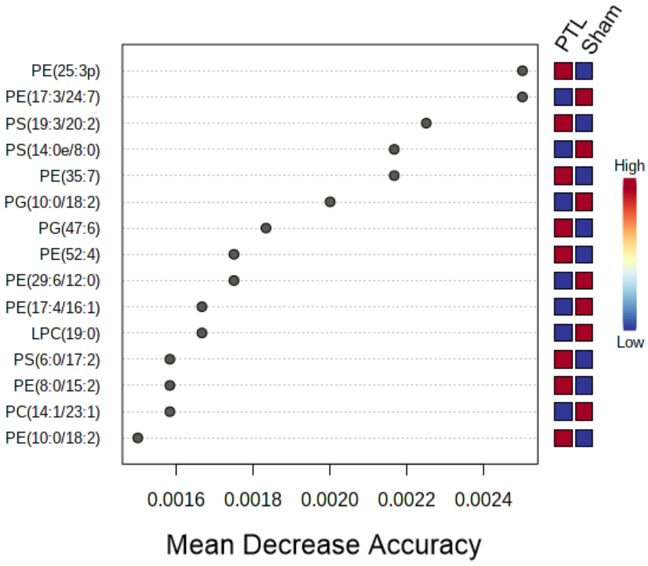

(A) **(B)**

Supplementary Figure S14: The volcano map showed the differential lipids expression level in the mice plasma of PTL vs PTL plus Tulsi groups (log2 FC= 1.5 and p-value <0.05 considered significant). Upregulated lipids are shown by red colour dots while downregulated lipids are shown by blue coloured dots.

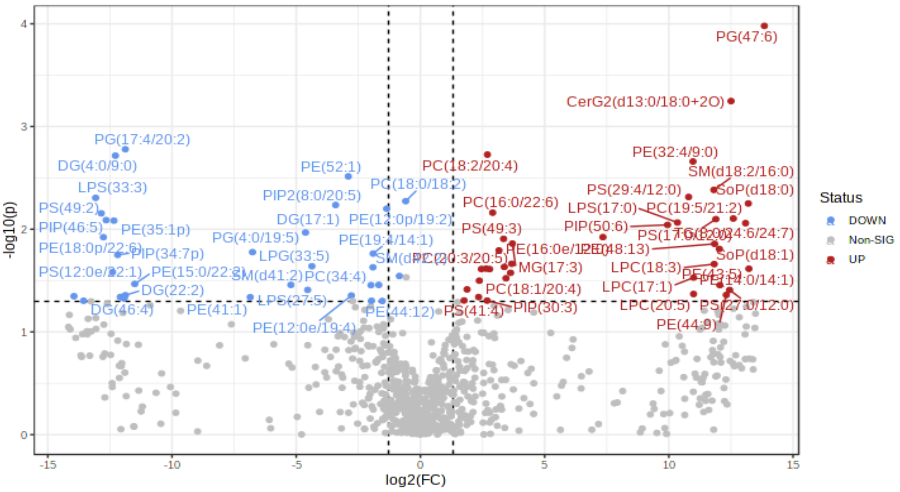

Supplementary Figure S15: (A) Partial Least Squares - Discriminant Analysis (PLS-DA) plot of lipid species in mice plasma of PTL vs PTL plus Tulsi groups revealing the component variance 27.4% and 18.5%. **(B)** VIP score of lipid species in PLS-DA in the mice plasma of PTL vs PTL plus Tulsi groups. The

coloured boxes on the right side represent the relative concentration of the corresponding lipid species. Red color indicates high and blue color indicates low.

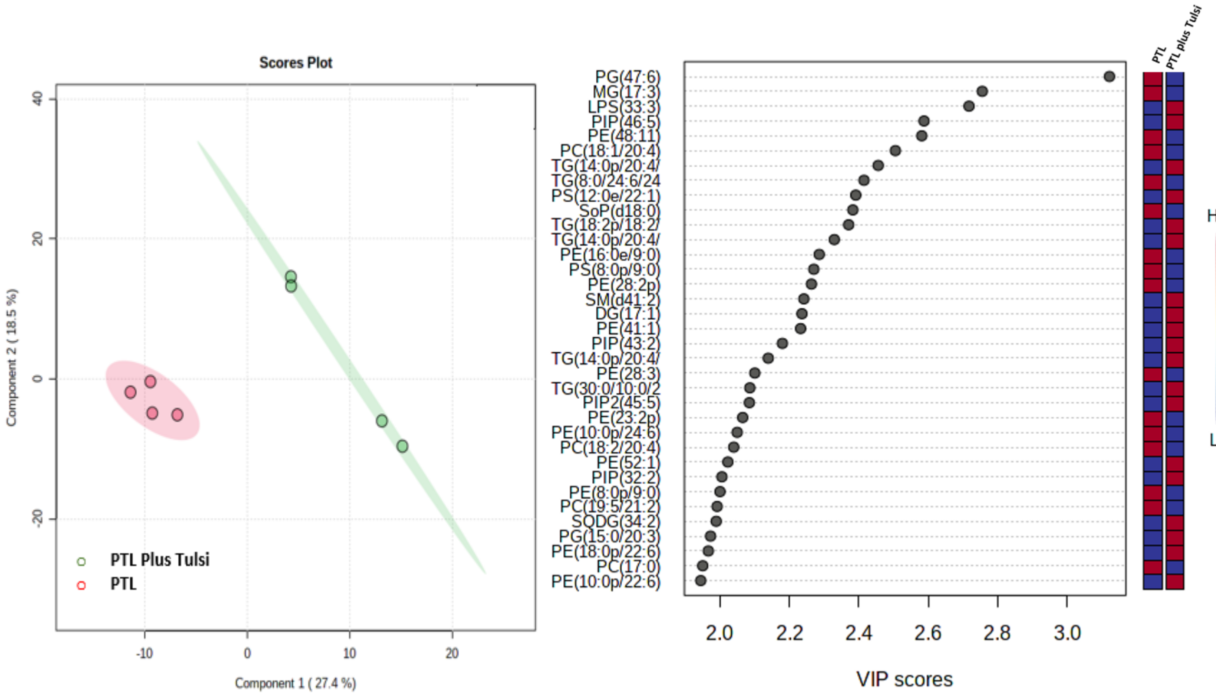

(A) **(B)**

Supplementary Figure S16: (A) Heatmap of the expression of identified differential lipids in the mice plasma between PTL vs PTL plus Tulsi groups. The coloured boxes on the right side represent the relative concentration of the corresponding lipid species. Red color indicates high and green color indicates low. **(B)** Random Forest from mice plasma of PTL vs PTL plus Tulsi groups identified 15 different lipid features and ranked by the mean decrease in classification accuracy.

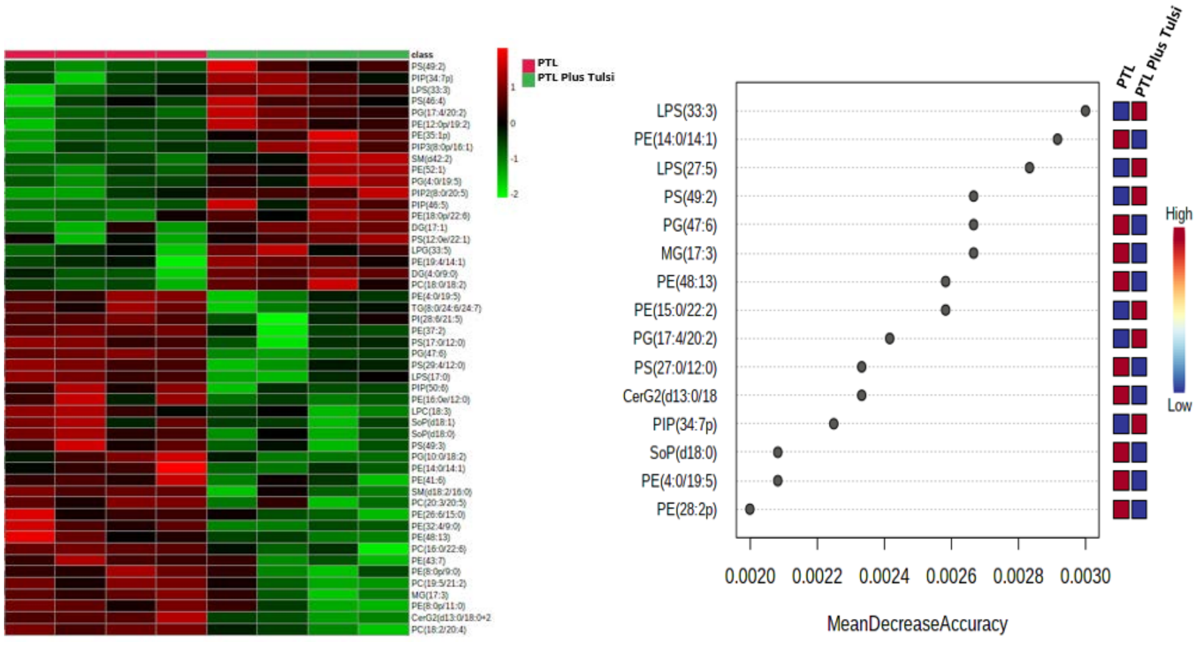

(A) **(B)**

Supplementary Figure S17: The volcano map showed the differential lipids expression level in the mice plasma of PTL vs PTL plus Ibu groups (log2 FC= 1.5 and p-value <0.05 considered significant). Upregulated lipids are shown by red colour dots while downregulated lipids are shown by blue coloured dots.

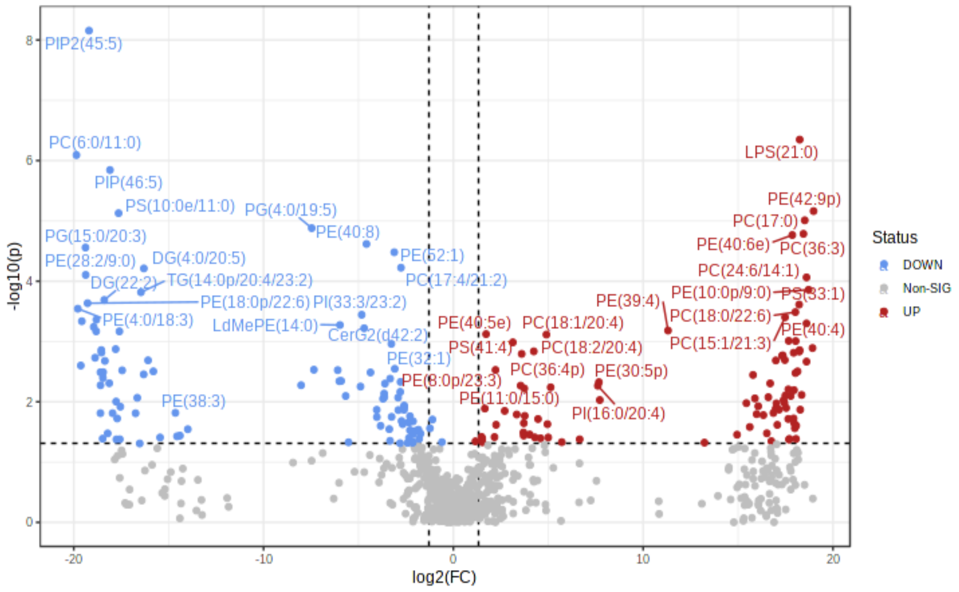

Supplementary Figure S18: (A) Partial Least Squares - Discriminant Analysis (PLS-DA) plot of lipid species in mice plasma of PTL vs PTL plus Ibu groups revealing the component variance 34.7%

and 17.7%. **(B)** VIP score of lipid species in PLS-DA in the mice plasma of PTL vs PTL plus Ibu groups. The coloured boxes on the right side represent the relative concentration of the corresponding lipid species. Red color indicates high and blue color indicates low.

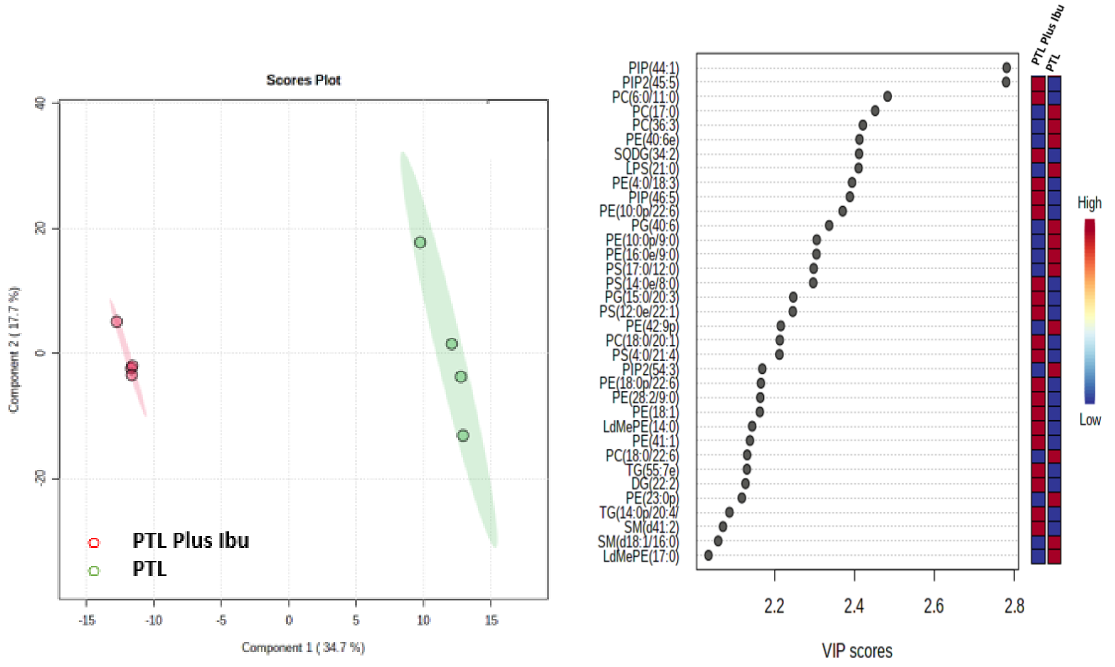

(A) **(B)**

Supplementary Figure S19: (A) Heatmap of the expression of identified differential lipids in the mice plasma between PTL vs PTL plus Ibu groups. The coloured boxes on the right side represent the relative concentration of the corresponding lipid species. Red color indicates high and green color indicates low. (**B)** Random Forest from mice plasma of PTL vs PTL plus Ibu groups identified 15 different lipid features and ranked by the mean decrease in classification accuracy.

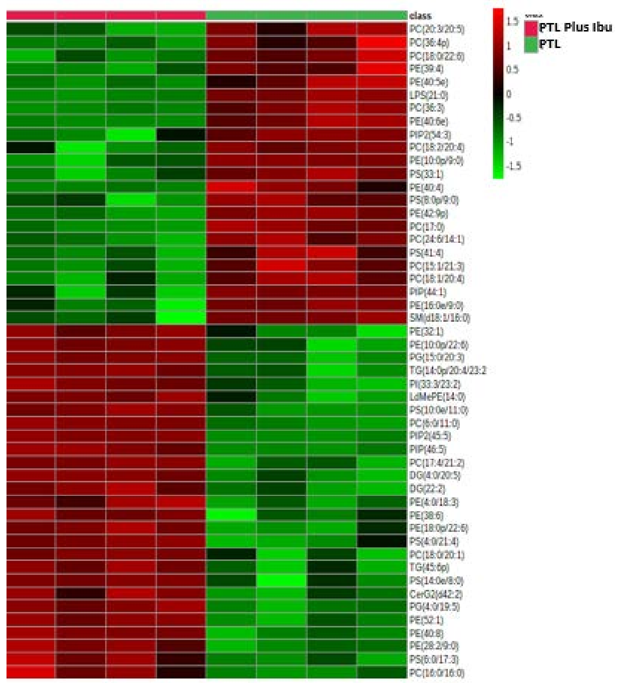
class
PC(20:3/20:5)
PC(36:4p)
PC(18:0/22:6)
PE(39:4)
PE(40:5e)
LPS(21:0)
PC(36:3)
PE(40:6e)
PIP2(54:3)
PC(18:2/20:4)
PE(10:0p/9:0)
PS(33:1)
PE(40:4)
PS(8:0p/9:0)
PE(42:9p)
PC(17:0)
PC(24:6/14:1)
PS(41:4)
PC(15:1/21:3)
PC(18:1/20:4)
PIP(44:1)
PE(16:0e/9:0)
SM(d18:1/16:0)
PE(32:1)
PE(10:0p/22:6)
PG(15:0/20:3)
TG(14:0p/20:4/23:2
PI(33:3/23:2)
LdMePE(14:0)
PS(10:0e/11:0)
PC(6:0/11:0)
PIP2(45:5)
PIP(46:5)
PC(17:4/21:2)
DG(4:0/20:5)
DG(22:2)
PE(4:0/18:3)
PE(38:6)
PE(18:0p/22:6)
PS(4:0/21:4)
PC(18:0/20:1)
TG(45:6p)
PS(14:0e/8:0)
CerG2(d42:2)
PG(4:0/19:5)
PE(52:1)
PE(40:8)
PE(28:2/9:0)
PS(6:0/17:3)
PC(16:0/16:0)
1.5
1
0.5
0
-0.5
-1
-1.5
PTL Plus Ibu
PTL

(A)

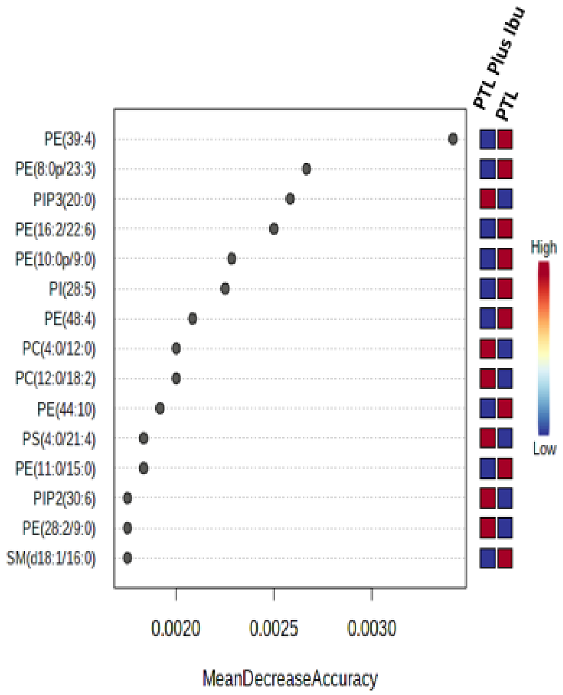
PTL Plus Ibu
PTL
PE(39:4)
PE(8:0p/23:3)
PIP3(20:0)
PE(16:2/22:6)
PE(10:0p/9:0)
PI(28:5)
PE(48:4)
PC(4:0/12:0)
PC(12:0/18:2)
PE(44:10)
PS(4:0/21:4)
PE(11:0/15:0)
PIP2(30:6)
PE(28:2/9:0)
SM(d18:1/16:0)
High
Low
0.0020
0.0025
0.0030
MeanDecreaseAccuracy

(B)
